# Laser-induced hyperspectral fluorescence for spatio-chemical detection of sunscreen contaminants in food-grade sea salt using sparse PCA–SVM analysis

**DOI:** 10.1007/s10653-026-03211-x

**Published:** 2026-05-05

**Authors:** Mohamed Ebrahem, Mohamed I. Hosni, Adel Abdallah, Alaaeldin Mahmoud

**Affiliations:** 1 Optoelectronics and Automatic Control Systems Department, Military Technical College, Cairo, Egypt; 2 Naval Warning Engineering Department, Military Technical College, Cairo, Egypt

**Keywords:** Laser-induced hyperspectral fluorescence, Sparse principal component analysis, Support vector machine, Sunscreen contaminants, Sea salt, Spatio-chemical mapping

## Abstract

Sea salt increasingly harbors organic contaminants from personal care products, yet current monitoring methods lack spatial resolution and require destructive sampling. This study introduces an innovative analytical framework integrating Laser-Induced Fluorescence (LIF) Hyperspectral Imaging (HSI) with machine learning for the rapid, non-destructive detection of sunscreen residues on salt crystals. To simulate contamination, seawater from the Mediterranean coast (Alexandria, Egypt) was spiked to achieve a 10 mg/L sunscreen concentration within the seawater matrix prior to crystallization; this formulation contained Ethylhexyl Methoxycinnamate, Homosalate, and Ethylhexyl Salicylate. A SOC710 HS camera (128 bands) acquired fluorescence data under 450 nm laser excitation. Raw data underwent preprocessing and dimensionality reduction via Sparse Principal Component Analysis (Sparse PCA, λ = 0.5, k = 4 components, 73.4% sparsity). A Support Vector Machine (SVM) with an RBF kernel was trained on these sparse features. Performance evaluation employed tenfold stratified cross-validation, an 80–20 holdout test on ROI-based spectra, and independent sample validation against manually annotated pixel-wise ground-truth masks. While ROI-based tests yielded near-perfect accuracy under ideal conditions, full-image evaluation achieved ≈96% pixel-wise accuracy (precision ≈ 0.99, recall ≈ 0.95, F1 ≈ 0.97), providing a realistic estimate under heterogeneous conditions. Full-image classification mapped widespread contamination (57.8% of pixels), whereas an independently prepared clean salt sample produced zero false positives. The integrated Sparse PCA–SVM framework transforms fluorescence-imaging data into spatio-chemical maps, simultaneously revealing contaminant presence and spatial distribution on salt surfaces, thereby offering a powerful paradigm for the interpretable monitoring of organic pollutants in food materials.

## Introduction

Sea salt remains one of the most widely consumed food additives globally, with yearly production exceeding 200 million tons (Geological Survey, 2024; Kalasariya & Pereira, 2025; Song et al., 2025; Vyas et al., 2022). Over one-third of, this volume is harvested through the solar evaporation of seawater. However, the fundamental mechanism of this process, osmotic concentration via progressive water removal, unintentionally concentrates dissolved anthropogenic pollutants alongside essential minerals (Caloni et al., 2021). These pollutants derive from several anthropogenic sources: municipal wastewater discharge, pharmaceutical residues, industrial effluent, and universally, residues from personal care products entering marine systems (Damikouka et al., 2024; Hodge et al., 2025; Lozano et al., 2020). Sunscreen chemicals rank among the most durable, biologically active, and geographically widespread pollutants identified in coastal salt producing regions (Bilal et al., 2020; Muvel et al., 2025). Essential chemicals such as Ethylhexyl Methoxycinnamate (EHMC), Homosalate and Ethylhexyl Salicylate serve as UV filters in commercial sunscreen formulations yet accumulate in marine ecosystems (Bordalo & Soares, 2025; Deng et al., 2025). When these compounds become integrated into salt crystals during crystallization, they are no longer subject to environmental degradation processes but become chemically confined within the crystalline matrix, protected from natural attenuation (Ciglova et al., 2024; Nieddu et al., 2024). Unlike acute exposures to contaminated water, salt consumption creates a chronic, low-dose exposure pathway. According to pooled data from the 2023 World Health Organization (WHO) global report, the estimated mean sodium intake is 2.5 g/day; this translates to approximately 6.4 g of salt per day, which already exceeds the WHO adult recommendation of < 5 g/day (WHO Global report on sodium intake reduction, 2023). Alarmingly, 73% of children are classified as having high sodium intake, suggesting that a substantial fraction of the global youth population experiences daily and lifelong exposure to any synthetic contaminants embedded within salt (An et al., 2024; Keshavarz et al., 2024). The problem is not geographically limited but rather most severe in coastal regions characterized by high tourism density, limited wastewater treatment infrastructure, or proximity to industrial activity (Pizzol et al., 2025). Current analytical practices for monitoring organic contaminants in sea salt are fundamentally inadequate for addressing this emerging risk. Traditional inspection approaches mandated by global food safety standards, such as visual examination for physical impurities, colorimetric evaluation for whitening agents, and argentometric titration (e.g., Mohr’s method) for chloride quantification, are primarily designed for bulk quality assurance and macro-composition analysis (Ward, 2024). While these methods are effective for ensuring minimum sodium chloride purity, they lack the chemical specificity and sensitivity required to identify trace organic compounds (Kaloo, et al., 2024; Nielsen, 2024). The current gold-standard laboratory techniques, Gas Chromatography–Mass Spectrometry (GC–MS) and Liquid Chromatography–Mass Spectrometry (LC–MS), provide outstanding analytical sensitivity with detection limits ranging from parts per trillion to parts per billion and deliver definitive compound identification (Picó & Campo, 2022). However, their deployment is severely restricted by multiple practical and scientific limitations:

*Destructive sampling* Both GC–MS and LC–MS require complete sample dissolution or extraction, destroying the original material and precluding any spatial analysis of contamination patterns (Grant et al., 2025).

*Time-intensive workflows* Sample preparation, including drying, grinding, extraction, and purification, in conjunction with instrumental analysis, generally requires 4–8 h per sample, hence constraining throughput and elevating analytical expenses (Nasri et al., 2025).

*Expertise requirements* The operation of GC–MS and LC–MS requires highly skilled specialists, restricting accessibility in resource-constrained environments and underdeveloped countries where contamination issues are most severe (Zhang et al., 2025).

While chromatographic techniques remain the gold standard for sensitivity, the food safety industry has increasingly adopted non-destructive optical methods for rapid screening. Recent state-of-the-art applications predominantly utilize HSI in the Visible-Near Infrared (Vis–NIR) diffuse reflection mode to detect adulterants in powdered foods and grains (Patel et al., 2024; Pu et al., 2023). However, these reflectance-based techniques struggle significantly with ‘white-on-white’ scenarios, where organic contaminants (like sunscreen residues) share similar scattering properties and visual appearance with the salt crystal matrix (Wang et al., 2024). Although Raman spectroscopy offers high molecular specificity for such identification, it is inherently limited by weak signal intensity and slow point-by-point acquisition speeds, rendering it impractical for high-throughput conveyor belt inspection (Li et al., 2024). Consequently, there is a critical technological gap for a method that combines the speed of wide-field imaging with the chemical specificity required to distinguish organic traces from an inorganic crystalline background. To address the limitations of the aforementioned methods, this study leverages the distinct photophysical properties of sunscreen residues. Sunscreen UV filters exhibit strong absorption in the UV-B region (310 nm) with characteristic fluorescence emission at 480–520 nm, creating a stark chemical contrast with salt, which exhibits negligible intrinsic fluorescence (Lorigo et al., 2024). Furthermore, transient absorption spectroscopy confirms that excited states of these filters generate long-lived absorption features spanning the visible region (350–675 nm), allowing for efficient detection upon 450 nm laser excitation (Lavorgna et al., 2024; Rajan, 2024). To capture these features, we integrate LIF with HSI approach. Unlike traditional single-point fluorescence, this approach generates a three-dimensional data cube (spatial dimensions X, Y plus the wavelength dimension), where each pixel contains a complete fluorescence spectrum (Okada et al., 2024; Saber et al., 2025a; Zhang et al., 2024a). This rich dataset allows for superior specificity, as multiple fluorescence bands can be simultaneously acquired to distinguish contamination from background noise and confirm chemical identity (Mahmoud et al., 2024; Saber et al., 2025b). Building on these optical principles, this study presents an innovative analytical framework integrating LIF with HSI for the rapid, non-destructive, and spatially resolved detection of sunscreen residues in sea salt. The primary technical contributions of this work are threefold:

1. *Spatio-chemical acquisition* We employ an orthogonal LIF-HSI configuration to simultaneously capture spectral signatures and spatial distribution patterns of contaminants, generating high-dimensional data cubes rich in chemical detail.
2. *Sparse feature extraction* To address the high dimensionality of spectral data (Ahmad et al., 2025; Wan et al., 2025), we utilize (Sparse PCA) with L_1_ regularization (Chang et al., 2024; Chen & Rohe, 2024; Lee et al., 2025; Murariu et al., 2025; Souza et al., 2024). This approach suppresses noise and isolates the most diagnostically relevant wavelength regions associated with sunscreen fluorescence, offering superior interpretability compared to conventional dimensionality reduction.
3. *Robust classification and validation* A SVM is trained on these sparse features to generate automated contamination maps (Afjal et al., 2024; Du et al., 2024; Gao et al., 2024; Kumar & Anand, 2024). Finally, to rigorously validate real-world performance, the resulting classification maps are evaluated against manually generated pixel-wise ground truth (GT) masks and independent uncontaminated samples, providing a realistic assessment of the method’s spatio-chemical mapping capabilities. Combining LIF-HSI with SVM classification and Sparse PCA dimensionality reduction creates a new analytical paradigm for quick, non-destructive food contaminant monitoring. Heterogeneous distribution patterns on salt crystal surfaces that would be entirely obscured in averaged bulk analysis are revealed by the demonstration of pixel-level contamination mapping. Finding the most discriminatory wavelength areas through the use of sparse feature selection allows for precise classification and a mechanistic comprehension of fluorescence signatures. The strong separability between contaminated and clean salt samples was verified through consistent performance across multiple validation schemes, including Region-of-Interest (ROI) based holdout testing, tenfold cross-validation, independent sample evaluation, and pixel-wise comparison against a manually annotated GT mask. The GT-based analysis yields a realistic estimate of ≈ 96% pixel wise accuracy under heterogeneous conditions. The developed workflow establishes a practical and scalable approach that can enable food safety regulators to rapidly and non-destructively screen sea salt originating from diverse geographical regions and production methods, thereby supporting proactive contamination monitoring and quality assurance.

## Material and methods

### Sample preparation and seawater crystallization

Seawater (aggregating approximately 1 L) was collected as a composite sample by combining aliquots from multiple high-traffic zones along the Mediterranean coast near Alexandria, Egypt. This composite sampling strategy ensures that the matrix captures the heterogeneous organic load and turbidity characteristic of heavily impacted marine environments. Crucially, the samples were used without pre-filtration or sterilization. This approach intentionally preserves the natural microbial community and dissolved pollutants present in the ecosystem, ensuring the ecological realism required to validate the method’s robustness against background interference. The collected seawater was divided into two identical 250 mL glass beakers and subjected to identical controlled experimental conditions for crystallization. Both beakers were maintained under ambient laboratory conditions (approximately 25 °C and 50 to 60 percent relative humidity) and manually agitated with a clean glass rod twice daily. A three-week crystallization timeframe was empirically established to ensure complete evaporative crystallization while allowing adequate time for constituent dispersion. To simulate realistic contamination in recreational beach waters, the first beaker was augmented with a commercial sunscreen formulation (SPF 50, broad-spectrum) at a concentration of 10 mg/L. The sunscreen formulation contained:

- *Primary UV-B filter* Ethylhexyl Methoxycinnamate (EHMC, also called octyl methoxycinnamate), serving as the dominant organic photo stabilizer.
- *Complementary UV filters* Homosalate and Ethylhexyl Salicylate, providing enhanced UV coverage.
- *Inorganic UV blocker* Titanium Dioxide (TiO_2_), included as a physical barrier.

**Fig. 1:**
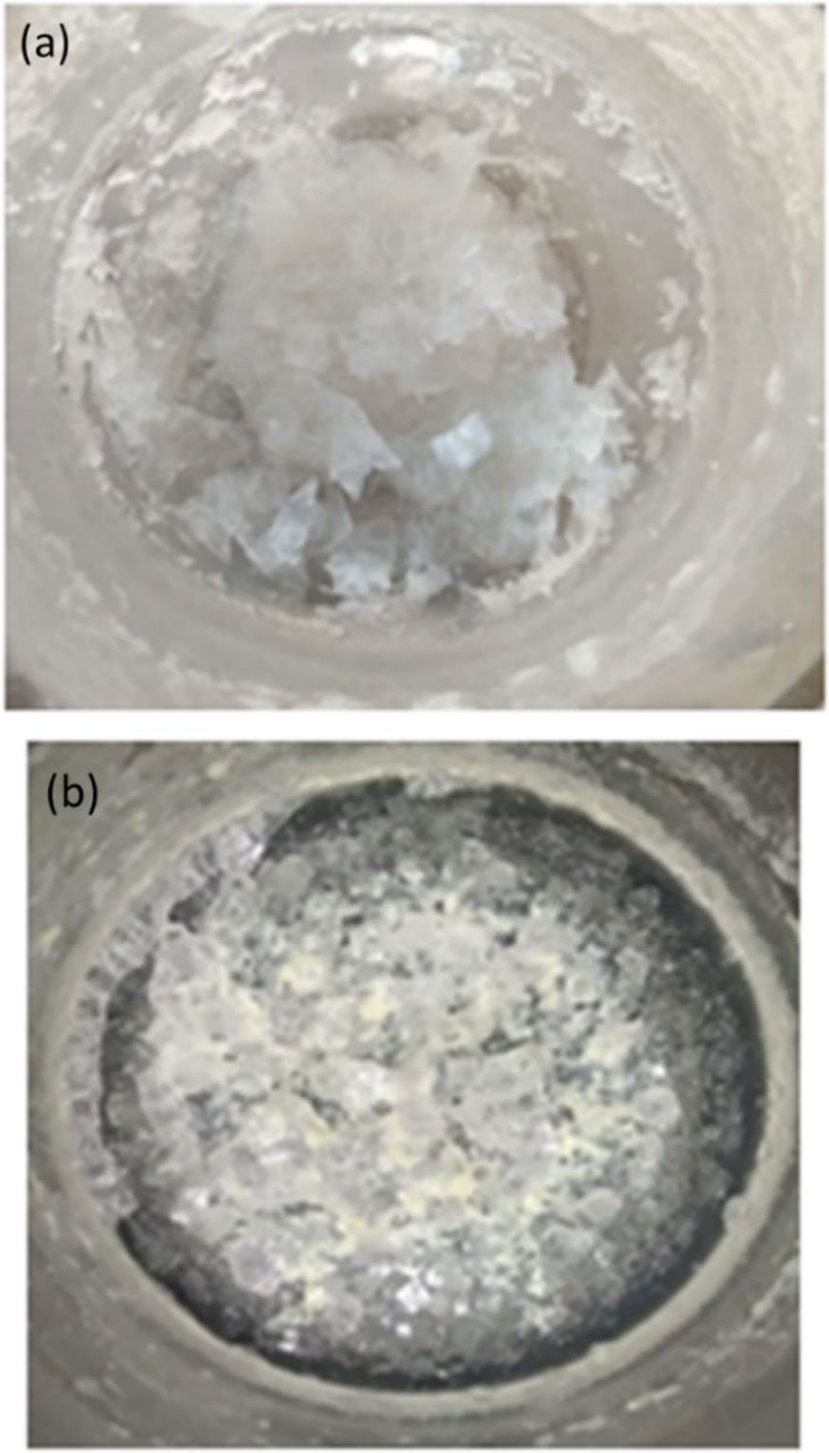
The physical characteristics of the crystallized samples show how sunscreen contamination manifested itself visually during the crystallization process. **a** The uncontaminated seawater crystallization **b** the sunscreen-contaminated seawater

Upon completion of the three-week period, this augmented sample produced salt crystals that were heterogeneously polluted with sunscreen residues (Fig. 1b). The second beaker served as the uncontaminated control. It received the exact same physical treatment and environmental exposure detailed above, with the intentional exclusion of the sunscreen formulation. This natural evaporative crystallization resulted in clean sea salt crystals devoid of synthetic organic impurities (Fig. 1a), functioning as the essential baseline reference for comparative spectral analysis.

As shown in Fig. 1a, The pure sodium chloride composition is reflected in the translucent to opaque white salt crystals produced by the uncontaminated seawater crystallization. However, because organic sunscreen ingredients (EHMC, Homosalate, and Ethylhexyl Salicylate) are incorporated into the crystal matrix, the sunscreen-contaminated seawater depicted in Fig. 1b yields salt crystals with heterogeneous coloration and surface characteristics. These physical observations provide preliminary visual evidence that sunscreen constituents are successfully incorporated into the crystallized salt structure, establishing the foundation for subsequent spectroscopic characterization.

### Hyperspectral imaging system and instrumentation

The SOC710 HSI camera was utilized as the primary analysis device. The SOC710 is a radiometrically calibrated push-broom spectrometer that incorporates a linear charge-coupled device (CCD) array paired with transmission diffraction grating spectroscopy, facilitating the concurrent collection of spatial (linear array, 1600 pixels) and spectral data (128 contiguous wavelength bands spanning 400—1000 nm at approximately 5.1 nm spectral resolution). Salt crystal specimens were lit using a continuous-wave blue laser source (λ_excitation_ = 450 nm, P = 50 mW, beam diameter ≈ 0.8 mm at the focal plane) situated at a working distance of 40 cm from the sample surface. The 450 nm wavelength was selected due to.

1. Ultrafast transient absorption spectroscopy revealing that sunscreen UV filters, such as EHMC and cinnamate derivatives, exhibit prolonged excited-state absorption and fluorescence characteristics extending into the blue-visible spectrum, significantly overlapping with 450 nm excitation.
2. Pure sodium chloride displaying minimal absorption and intrinsic fluorescence at this wavelength, thereby ensuring a low background signal and high contrast.
3. 450 nm being outside the predominant band of ambient visible light, which diminishes interference from environmental sources while efficiently exciting photoactive sunscreen residues.

**Fig. 2:**
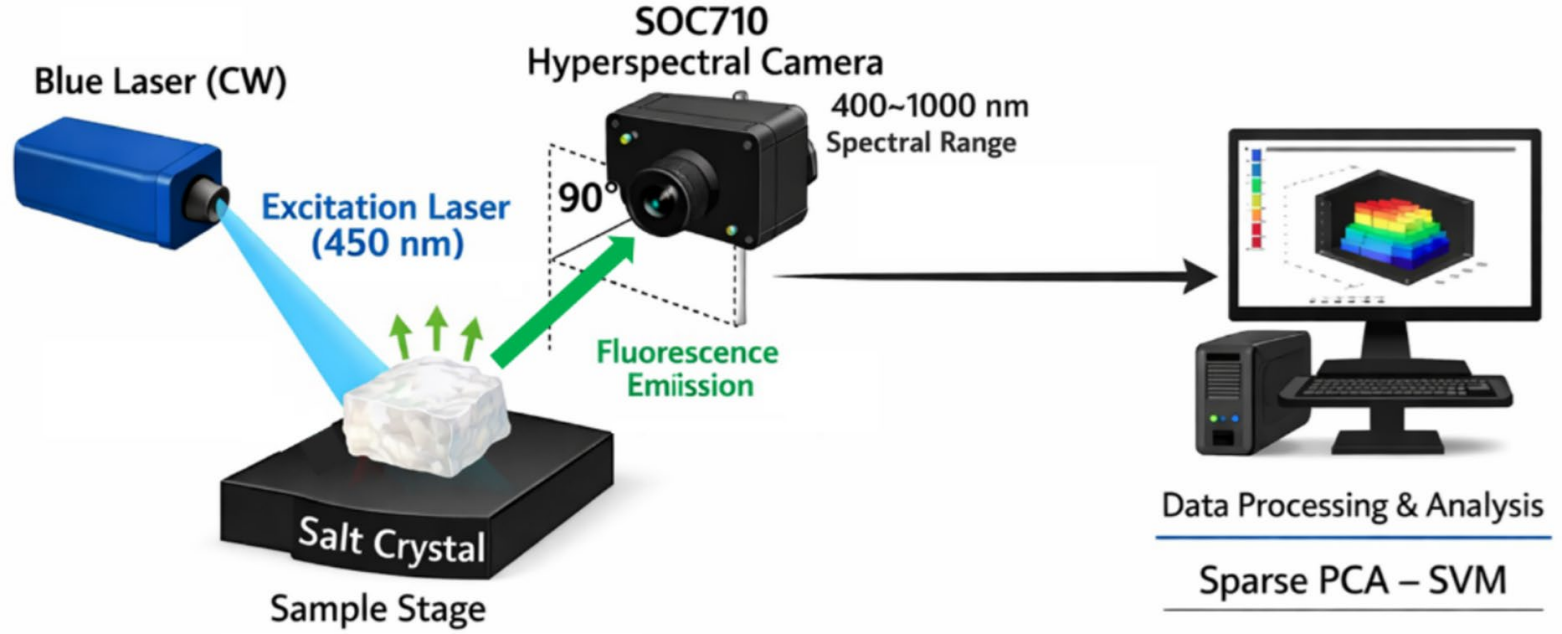
Schematic diagram of the LIF-HSI experimental setup. The system utilizes a continuous-wave 450 nm blue laser for excitation, positioned at a 90° angle relative to the optical axis of the SOC710 hyperspectral camera. This orthogonal geometry minimizes specular reflection while maximizing the collection of isotropic fluorescence emission from the salt crystal surface. The hyperspectral camera captures the spatial and spectral data (400–1000 nm), which is transferred to a workstation for preprocessing and Sparse PCA–SVM analysis

The HS camera was oriented perpendicularly, or at a precise 90° angle to the incident laser beams illustrated in Fig. 2. This orthogonal configuration maximizes the detection of isotropic fluorescence emission, which radiates uniformly in all directions from excited fluorophores, while minimizing the collection of specular laser scatter and Rayleigh-scattered light (Ebrahem et al., 2026).

As shown in Fig. 2, this setup allows for the simultaneous acquisition of spatial and spectral information across salt crystal samples. HS data cubes are then processed through software-based preprocessing, calibration, feature extraction (sparse PCA), and machine learning classification (SVM).

### Radiometric calibration procedure

Before all measurements, a two-point radiometric calibration was conducted to address sensor noise, non-uniform light, and dark current.

*White reference acquisition* A Spectralon® diffuse reflectance standard panel (99% reflectivity from 400 to 1000 nm) was positioned at the sample location under the same laser illumination geometry. The resultant hyperspectral image functioned as the benchmark for optimal signal.

*Dark reference acquisition* An opaque, non-reflective cover was positioned directly over the camera lens, obstructing all incoming light. The resultant image captured the camera’s baseline electrical noise and dark current throughout all spectral bands.

*Radiometric correction* Raw HS data (*R*_*o*_) for each sample were corrected using the following equation (Mahmoud & El-Sharkawy, 2024):

1 $${R}_{f}=\frac{{R}_{o}-{R}_{D}}{{R}_{B}-{R}_{D}}$$

where *R*_*f*_ represents the calibrated image, *R*_*o*_ is the original raw image of the sample, *R*_*D*_ is the dark reference image, and *R*_*B*_ is the bright (white reference) image. This adjustment removes systematic errors and normalizes the raw pixel intensities to physical reflectance units (0–1 range).

### Region of interest selection strategy

**Fig. 3:**
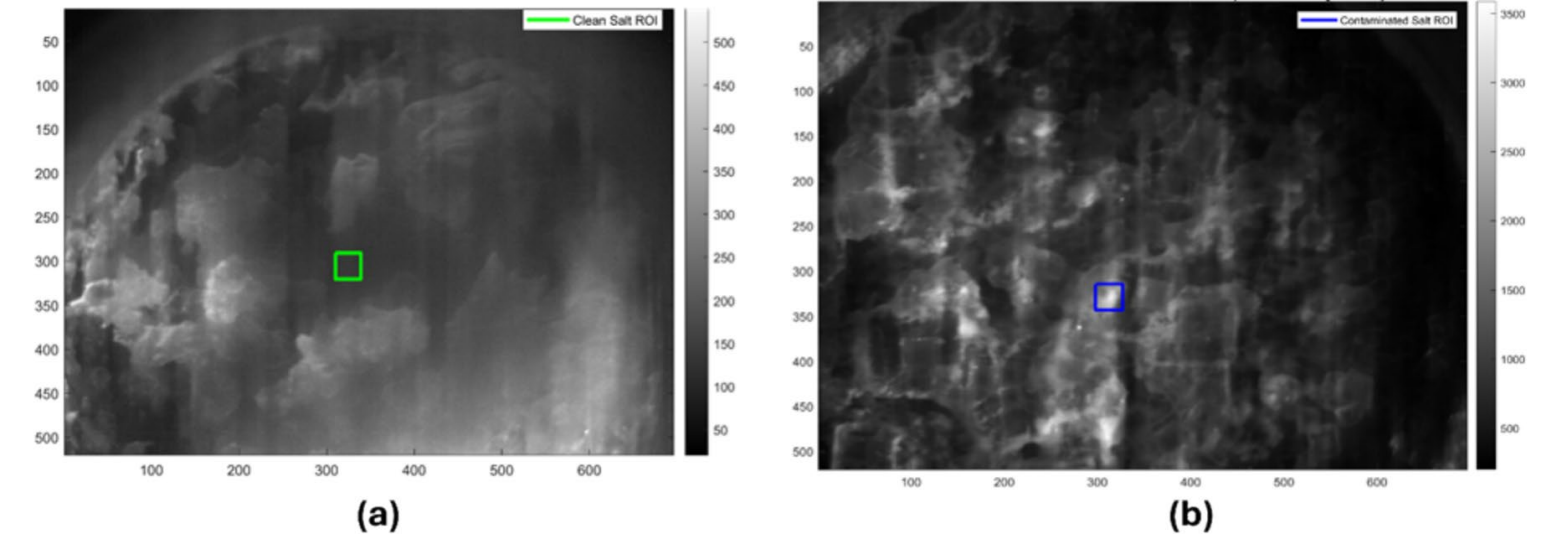
The HS image in gray format (displaying HS band 23 at approximately 512 nm). **a** ROI selection in the clean salt cube (green box); **b** ROI selection in the sunscreen contaminated salt cube (blue box)

A novel fixed-size ROI selection approach was employed to mitigate sampling bias and guarantee reproducibility:

- *ROI size definition* Dimensions of 30 × 30 pixels (totaling 900 pixels per ROI) were standardized to provide uniform sampling across all analyses.
- *ROI placement* Two ROIs were selected on monochromatic HS images, as shown in Fig. 3:
- *Salt crystal ROI* Located at pixel coordinates (339, 300) in the uncontaminated salt cube.
- *Sunscreen residue ROI* Located at pixel coordinates (310, 320) in the contaminated salt cube.
- *Equal class representation* In order to guarantee balanced class representation in the training dataset, both ROIs had the same number of pixels (900 each).

To guarantee statistical robustness, HS measurements were conducted ten times for each sample. During consecutive acquisitions, samples were temporarily extracted from the illumination field and realigned to accommodate minor positional discrepancies (≤ 2 mm). During all measurements, the laser source and camera were positioned immovably to ensure total consistency in illumination and detection geometry, which is essential for accurate fluorescence intensity quantification.

As shown in Fig. 3, two fixed 30 × 30-pixel ROIs are overlaid as colored boundary boxes: the green-bordered rectangle demarcates the clean salt crystal region at coordinates (339, 300), while the blue-bordered rectangle identifies the sunscreen-contaminated region at coordinates (310, 320). This standardized ROI placement ensures reproducible sampling and equal class representation for model training and validation.

### Spectral preprocessing pipeline

A systematic, multi-step preprocessing procedure was applied to raw HS cubes:

- Step 1: Laser band removal.

The spectral bands within the 400–480 nm range, associated with the laser excitation wavelength and its adjacent spectrum, are significantly compromised by laser scatter and Rayleigh scattering. These bands introduce extraneous signals unrelated to sample fluorescence, compromising subsequent analysis. These bands (17 out of 128 total) were methodically eliminated, leaving 111 bands between 480 and 1000 nm for additional examination.

- Step2: Baseline correction.

Due to temperature changes, dark current drift, and weak broadband autofluorescence from salt crystal lattice defects, fluorescence spectra frequently show slowly changing baseline offsets. The 10th percentile intensity value for each pixel spectrum was calculated at every wavelength and deducted from the total spectrum. This method successfully eliminates additive background while maintaining the tiny fluorescence emission peaks’ structural integrity.

- Step 3: Negative value clipping.

By their very nature, fluorescence intensities are not negative. To preserve physical validity, negative values resulting from noise or overcorrection were set to zero.

- Step 4: Area normalization.

To account for variations in total fluorescence intensity (due to differences in sunscreen concentration, crystal surface roughness, or minor illumination variations), each spectrum was normalized by its integrated area under the curve (Peng et al., 2023):

2 $${I}_{norm\left(\lambda \right)}=\frac{{I}_{corrected\left(\lambda \right)}}{{\int}_{\lambda 1}^{\lambda 2}{I}_{corrected\left({\lambda }{\prime}\right)d{\lambda }{\prime}}}$$

where *λ*_*1*_ and *λ*_*2*_ represent the lower and upper limits of the effective spectral integration range, explicitly defined as 480 nm and 1000 nm, respectively. By eliminating absolute intensity changes and maintaining spectral shape (the relative distribution of emission across wavelengths), this normalization increases the robustness of downstream classification.

- Step 5: Savitzky-Golay smoothing.

A Savitzky-Golay finite impulse response (FIR) filter (polynomial order: 3, window length: 11 points) was then used to smooth each spectrum. Compared to ordinary moving-average filters, this filter successfully attenuates high-frequency noise while maintaining peak positions, heights, and widths by fitting a polynomial to a moving window of spectrum values.

### Sparse principal component analysis (PCA)

Conventional PCA maximizes variance retention through orthogonal transformations, but produces dense loading vectors spanning all original variables (Rodionova et al., 2021). When analyzing high-dimensional spectral data (111 bands) with limited training samples, dense PCA features are susceptible to the “curse of dimensionality” and often include noise-driven components lacking physical interpretation. Sparse PCA addresses this limitation by incorporating explicit L_1_ (Lasso) regularization (Guerra-Urzola et al., 2025). This technique maximizes the variance explained by each component while simultaneously applying a penalty on the absolute size of its coefficients. The strength of this sparsity penalty, controlled by the parameter λ, drives many of the loading vector coefficients to exactly zero. The result is a set of components where the non-zero loadings are concentrated only in physically significant wavelength regions. The proposed algorithm was implemented in MATLAB (R2023b) for a centered data matrix X ∈ R^n×p^, the algorithm iteratively solves for the sparse loading vector$v$. The computation follows a power-iteration scheme. A static soft-thresholding operator is commonly used in standard Sparse PCA formulations. Nevertheless, when high-intensity excitation peaks are present, this strategy risks suppressing chemically relevant but low-intensity secondary fluorescence bands. To solve this, our implementation introduces an adaptive relative thresholding mechanism (Chen & Rohe, 2024; Lee et al., 2025).

For each component, the update step is defined as:

3 $$z = X^{{\mathrm{T}}} \left( {X v_{old} } \right)$$

To enforce sparsity, an adaptive soft-thresholding operator $\mathcal{S}$ is applied to the proxy vector ${\boldsymbol{z}}$:

4 $${\boldsymbol{v}}new=\frac{\mathcal{S}\left({\boldsymbol{z}},\tau \right)}{||\mathcal{S}\left({\boldsymbol{z}},\tau \right){||}_{2}}$$

where the threshold $\tau$ is dynamically scaled relative to the maximum signal magnitude to ensure consistent sparsity across components with varying variance contributions (Zhang et al., 2024b):

5 $$\tau = \lambda \cdot max\left(\left| {\boldsymbol{z}}\right|\right)$$

Here, $\lambda$ is the user-defined sparsity parameter. The function $\mathcal{S}\left({\boldsymbol{z}},\tau \right)$ sets coefficients with absolute values below $\tau$ to zero, effectively selecting only the most diagnostic wavelengths:

6 $$S\left({z}_{j}, \tau \right)= sign\left({z}_{j}\right)\cdot \mathrm{max}\left(\left|{z}_{j}\right|- \tau , 0\right)$$

Following the convergence of the first component, the data matrix is deflated using Hotelling’s deflation to remove the variance explained by the current component before calculating the next (Liu et al., 2026):

7 $$X_{next} = X - \left( {X v} \right)v^{{\mathrm{T}}}$$

Based on the optimization described in “Hyperparameter optimization” section, we selected $k=4$ components and a sparsity parameter $\lambda = 0.5$. This configuration resulted in sparse loading vectors where non-zero coefficients were strictly confined to spectral bands associated with the fluorescence of sunscreen residues, effectively suppressing noise and irrelevant spectral background.

### Support vector machine (SVM) classification

SVM tackle binary classification by identifying the best possible separating boundary between two classes (Kusunoki & Tatsumi, 2025). This “optimal hyperplane” is chosen specifically to maximize the margin, which is the empty space between the boundary and the closest data points from each class. This approach also works to minimize misclassifications. For perfectly separable data, the “hard-margin” SVM finds the single boundary with the widest possible margin (Maggioni & Spinelli, 2025). However, real-world data often has overlap, so the “soft-margin” SVM is used. The $fitcsvm$ function in the MATLAB Statistics and Machine Learning Toolbox (R2023b) was used in this work to create the classification model. This method allows for a few data points to be on the wrong side of the margin in a controlled way (Zhang & Yang, 2025). A key parameter, the box constraint C, manages this trade-off, determining whether to prioritize a wider margin or perfect training accuracy was set to $C = 1$. The key theoretical strength of SVMs lies in the Structural Risk Minimization principle. Unlike models that focus solely on fitting the training data, SVMs are designed to find a simple and effective model that is more likely to perform well on new, unseen data (Roy & Chakraborty, 2023). This is achieved by optimizing for a wide margin, which inherently controls the model’s complexity and provides a theoretical guarantee for its generalizing ability, regardless of the number of features. This guarantee is crucial in our case, where the number of training samples is similar to the number of effective features after data processing. The Radial Basis Function (RBF) kernel was selected for its ability to handle complex, non-linear relationships, with the kernel scale ($\sigma$) set to auto so that heuristic is automatically optimized using the training set. This kernel works by implicitly transforming the features into a very high-dimensional space where a curved boundary in the original data can be treated as a straight line (Roman et al., 2021). Critically, predictor standardization was enabled inside the model architecture to ensure that input sparse scores were scaled to unit variance, preventing features with larger numerical magnitudes from skewing the optimization. This allows the SVM to apply its powerful margin-maximization principle to these complex problems (Lai et al., 2023). SVM approach was selected as the recognition model due to its demonstrated superiority in chemometric applications involving high-dimensional spectral data with limited training samples. Unlike deep learning approaches, which are prone to overfitting when labeled data is scarce, or simple and instance-based classifiers such as Linear Discriminant Analysis (LDA) and k-Nearest Neighbors (k-NN), which struggle to model complex spectral nonlinearities, SVMs leverage the kernel trick to establish robust decision boundaries. This makes SVM the optimal choice for ensuring generalization capabilities on unseen salt samples.

To ensure the rigor of the classification framework, a theoretical performance evaluation was conducted comparing the proposed SVM approach with two other standard chemometric classifiers, namely LDA and Random Forest (RF). The selection of SVM is justified by the specific constraints of the dataset, specifically high spectral collinearity and a limited number of validation samples. As detailed in Table 1, recent literature confirms that while LDA and RF are effective for general datasets, they exhibit critical limitations when applied to high-dimensional spectral imaging.

**Table 1:** Comparative analysis of classifier suitability for Laser-Induced Hyperspectral Fluorescence data

Algorithm	Key limitation for HS data	Suitability for this study
Linear discriminant analysis (LDA)	Linearity constraint and singularity 1. *Singularity* on raw HS data, high multicollinearity makes the covariance matrix non-invertible 2. *Linearity* Assumes classes are linearly separable with equal covariance	Linear classifiers are fundamentally inadequate for resolving the highly non-linear decision boundaries generated by complex multiple scattering within the heterogeneous crystalline matrix. (Medina-García, et al., 2025; Zhao et al., 2024)
Random forest (RF)	Data hunger & overfitting Builds decision boundaries by averaging many deep decision trees. Recent studies indicate it struggles to generalize on small datasets (limited ground truth pixels) and is computationally slower than SVM during real-time inference	In highly scattering granular matrices, RF requires massive training datasets to prevent overfitting. On limited samples, it exhibits higher variance, reduced spatial generalization, and lower overall accuracy compared to optimal-margin classifiers. (Fife & D’Onofrio, 2023; Raparthi, et al., 2024; Serranti et al., 2024)
Support vector machine (SVM)	None (optimal) Uses the ‘Kernel Trick’ to map non-linear spectral data into a higher-dimensional space where it is linearly separable. It relies only on support vectors (boundary points), making it robust against the ‘Curse of Dimensionality’.	Optimizes the hyperplane decision margin between complex spectral classes, effectively mitigating the curse of dimensionality to ensure robust spatial mapping despite limited training data. (Choi, et al., 2025; Haut et al., 2024)

### Ground truth extraction

**Fig. 4:**
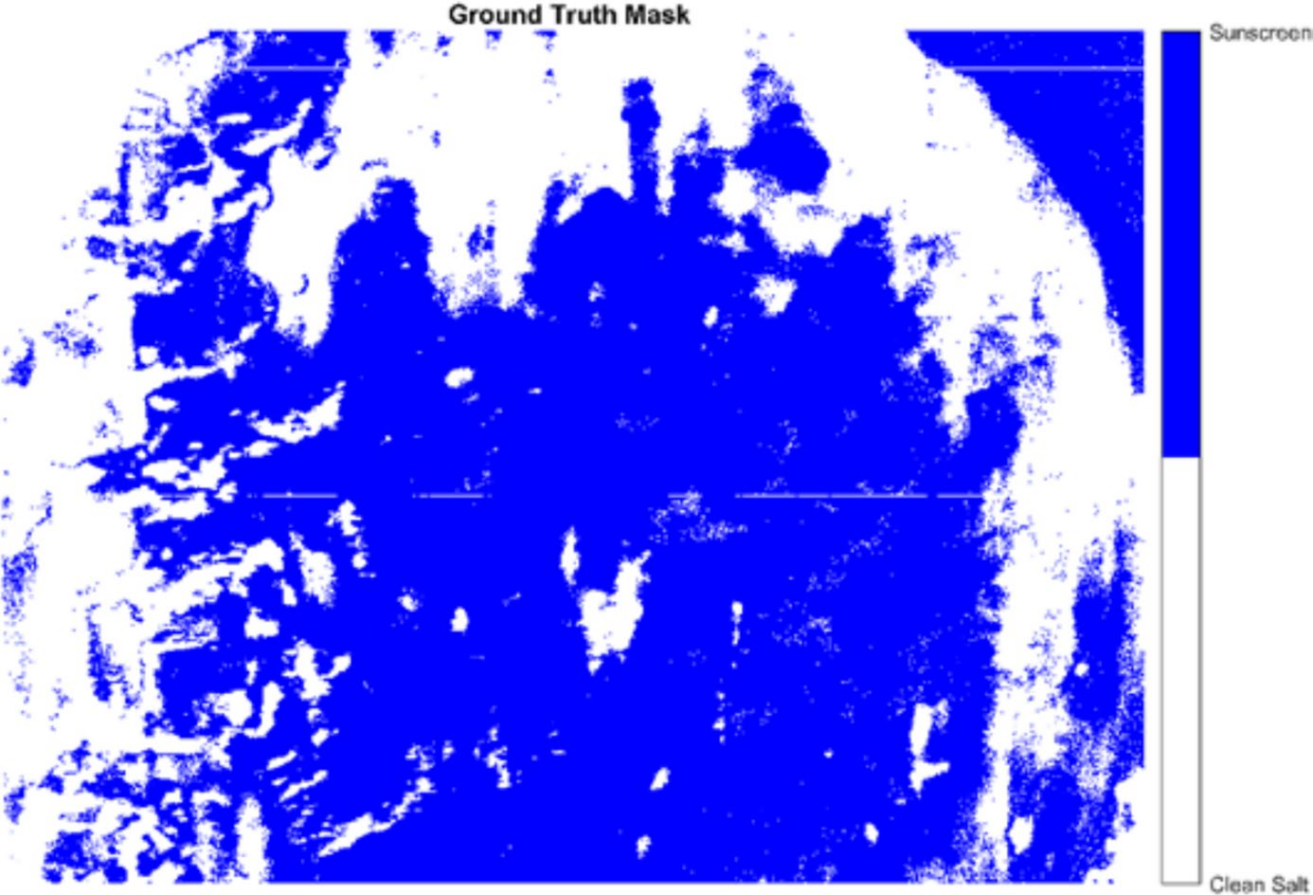
Ground truth mask for the sunscreen contaminated (blue) regions and salt crystal (white) regions

To completely evaluate the spatial classification performance beyond basic spectrum cross-validation, a GT mask was generated using a semi-automated annotation interface in MATLAB. To ensure the reliability of this reference data, a critical prerequisite for robust machine learning validation, a three-stage quality assurance protocol was employed:

- *High-contrast segmentation* Initial regions were identified on the pseudo-color representation of band 23 (~ 512 nm), where the fluorescence contrast between the sunscreen pollutants and the non-fluorescent salt background is maximally distinct. The resulting binary ground truth mask, which designates contaminated areas and pure salt regions, is illustrated in Fig. 4.
- *Spectral verification* To prevent subjective labeling errors, a random subset of 100 pixels from each designated class was inspected spectroscopically. Only regions where pixels consistently exhibited the characteristic fluorescence peak (for contaminated class) or a flat baseline (for clean class) were retained.
- *Boundary exclusion* To eliminate the uncertainty associated with ‘mixed pixels’ at the interfaces between crystals and contaminants, a morphological erosion operation (1-pixel radius) was applied to the mask boundaries.

This rigorous filtering yielded a high-confidence validation set. From this refined mask, we derived a balanced subset of 4000 randomly selected pixels (2000 contaminated and 2000 clean) to facilitate rigorous statistical analysis, ensuring that the ground truth represents ‘pure’ spectral endmembers rather than ambiguous edge artifacts. The resulting mask visualizes the accumulation of sunscreen contaminants (blue regions) on the salt surface (white regions). This ground truth functioned as the definitive reference for evaluating the pixel- wise predictions produced by the SVM model. These GT labels were not utilized during the model training phase; they functioned solely as an independent reference for the performance evaluation outlined in “Support vector machine classification performance” section.

### Performance metrics

Performance was measured by doing a thorough pixel-wise comparison between the independent GT mask established in “Ground truth extraction” section and the SVM-generated classification map in order to evaluate the model’s dependability in a realistic monitoring environment. This spatial validation tests the model’s capacity to precisely identify contamination patterns over the whole crystal surface, in contrast to normal cross-validation, which evaluates discrete spectral spots. Classification performance was quantified using Standard machine learning metrics (Aboalia et al., 2025):

8 $${\mathrm{Accuracy}} = \left( {{\text{TP }} + {\text{ TN}}} \right)/\left( {{\text{TP }} + {\text{ TN }} + {\text{ FP }} + {\text{ FN}}} \right)$$

9 $${\mathrm{Precision}} = {\mathrm{TP}}/\left( {{\text{TP }} + {\text{ FP}}} \right)$$

10 $${\mathrm{Recall}} = {\mathrm{TP}}/\left( {{\text{TP }} + {\text{ FN}}} \right)$$

11 $${\mathrm{F1}} - {\mathrm{score}} = {2} \times \left( {{\mathrm{Precision}} \times {\mathrm{Recall}}} \right)/\left( {{\mathrm{Precision}} + {\mathrm{Recall}}} \right)$$

- *Confusion Matrix* Where TP = true positives, TN = true negatives, FP = false positives, FN = false negatives.

Subsequent to model validation, the trained SVM was utilized on each pixel of the complete HS image (361,920 pixels, dimensions 520 × 696) to produce an exhaustive contamination map. Pixel-wise predictions were reformatted into a two-dimensional image and represented using color coding: green for clean salt (class 0) and blue for sunscreen-contaminated salt (class 1).

## Experimental results and analysis

### Analysis of spectral fluorescence response and scale transformation

To thoroughly assess the efficacy of the proposed HS fluorescence imaging technique in distinguishing between uncontaminated and sunscreen-contaminated sea salt, we initially analyzed the spectral fluorescence responses of salt samples under blue laser excitation (λ = 450 nm). The LIF technique effectively probes the molecular fingerprints of integrated sunscreen components that are undetectable by traditional imaging, thus presenting a distinctive method for contamination detection.

#### Linear scale spectral analysis

**Fig. 5:**
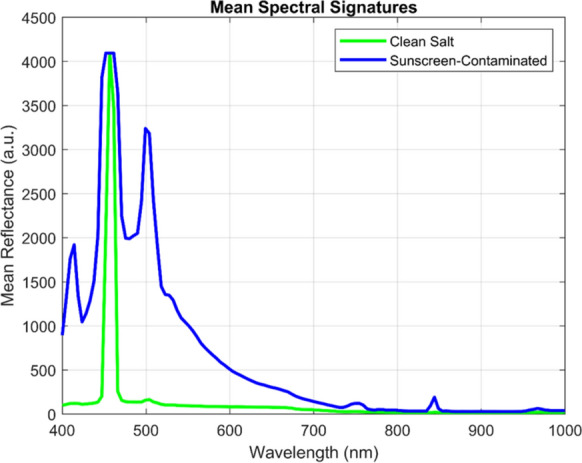
Average raw LIF intensity curves for salt crystal and sunscreen residues (10 mg/L) under blue laser (450 nm) excitation

Fluorescence spectra were preprocessed for both uncontaminated and sunscreen-contaminated (10 mg/L) salt samples. Figure 5 illustrates the mean spectrum fluorescence intensity profiles adjusted to the maximum recorded intensity across all samples, depicted on a standard linear intensity scale (arbitrary units).

As depicted in Fig. 5, The linear-scale representation reveals differences in spectral signature between the two sample types. The sunscreen-contaminated sample (blue line) exhibits a dominant fluorescence peak after the excitation laser source at approximately 480—520 nm. This peak corresponds to the characteristic fluorescence emission of EHMC, the primary UV filter present in the sunscreen formulation. In contrast, the clean salt sample in (green line) displays a characteristically flat spectrum, exhibiting only faint reflection of the laser excitation source (~ 450 nm) and relatively low background fluorescence (~ 200–400 counts) distributed uniformly across the spectral window.

#### Logarithmic (dB) scale transformation

**Fig. 6:**
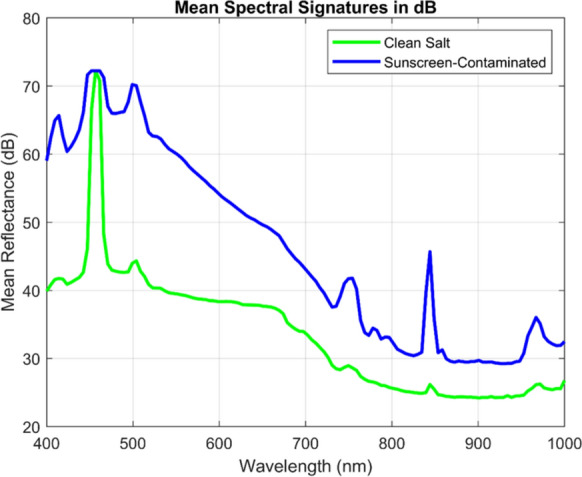
Average normalized LIF intensity curves expressed in decibels (dB) salt crystal and sunscreen residues (10 mg/L) under blue laser (450 nm) excitation for highlighting relative emission strengths and key spectral differences used for samples discrimination

To address the graphical constraints of linear-scale representation while preserving quantitative precision, we employed a logarithmic transformation to convert linear-scale intensities to the decibel (dB) scale. The transformation employs the amplitude-based formula (Weng & Coulter, 2025):

12 $${R}_{dB}=20{\mathrm{log}}_{10}\left({R}_{linear}\right)$$

where ${R}_{Linear}$ is the linear-scale intensity and the factor of 20 (rather than 10) is appropriate for amplitude-based optical measurements, following established signal processing standards. Figure 6 displays the identical spectral data from Fig. 5, now transformed to logarithmic dB scale.

The logarithmic transformation dramatically improves spectral feature visibility. The linear-scale 4000:1 intensity ratio (4100 counts vs. 300 counts) compresses to approximately 72 dB, transforming the axis from a compression problem to an expansion opportunity. Plotting the spectral fingerprints in dB makes it easier to identify areas with maximal contrast for material classification by highlighting even small differences between contaminated and clean salt across wavelengths. The multi-wavelength spectral complexity revealed in dB-scale visualization directly informs and validates the sparse PCA analysis presented in “Sparse principal component analysis” section. Sparse PCA will identify those components 2–4 concentrate their non-zero loadings approximately at these wavelength regions, demonstrating that mathematical feature extraction algorithms independently discover the same meaningful spectral signatures that become visually obvious only in dB-scale representation. Strong evidence that the identified features are actual spectroscopic characteristics of sunscreen contamination rather than data processing artifacts is provided by the fact that both unsupervised machine learning (sparse PCA) and human visual inspection (dB scale) independently identify the same wavelength regions as diagnostically significant.

#### Preprocessing effects: spectral refinement through pipeline

**Fig. 7:**
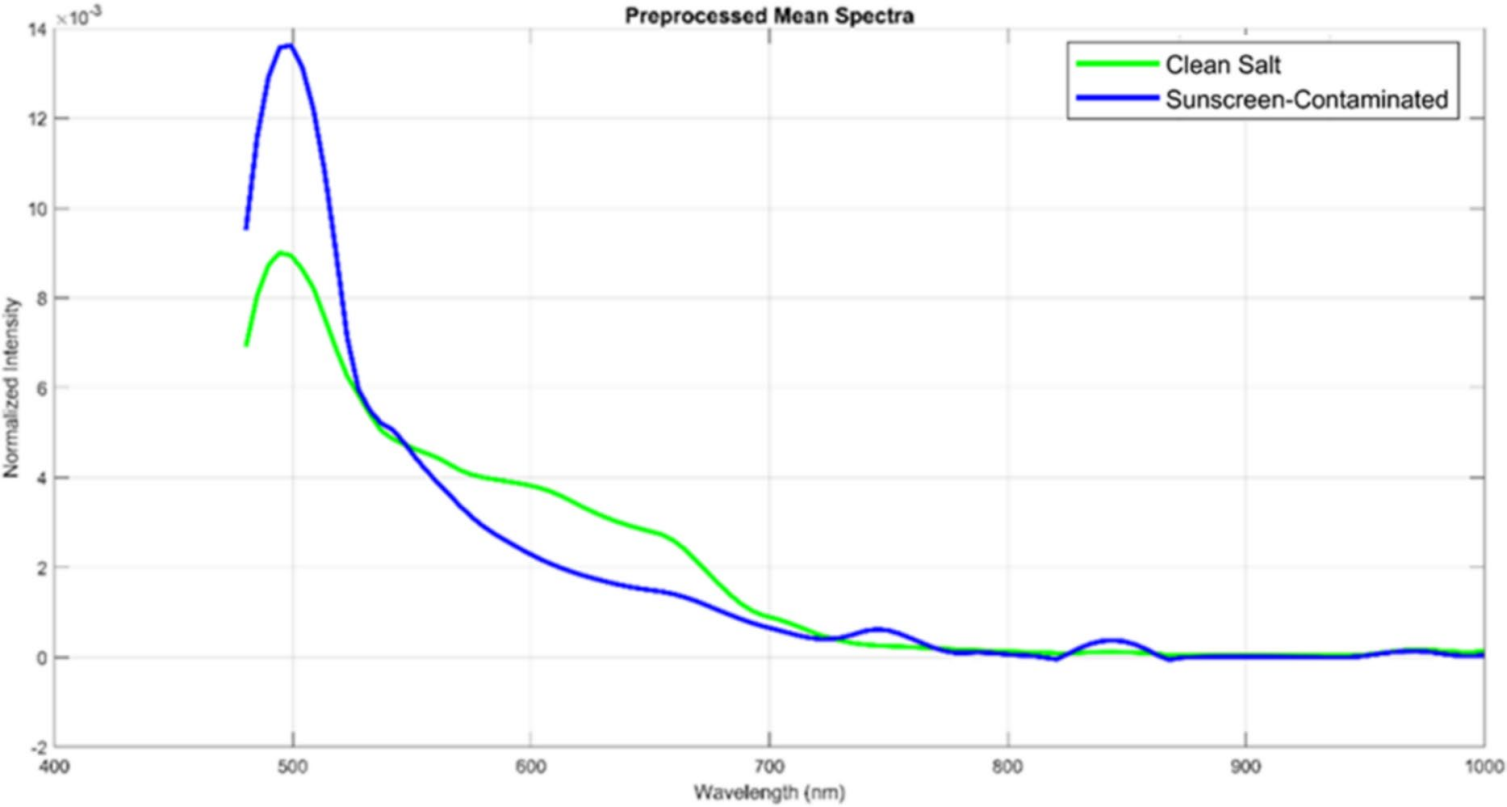
Mean normalized laser-induced fluorescence intensity spectra for salt crystal (green line) and sunscreen residues (10 mg/L) (blue line) after complete preprocessing pipeline (laser band removal, baseline correction, negative value clipping, area normalization, and Savitzky-Golay smoothing)

To demonstrate the effectiveness of the five-step preprocessing pipeline described in “Spectral preprocessing pipeline” section, the spectra were processed through the following systematic refinement steps: laser band removal (400–480 nm), baseline correction, negative value clipping, area normalization, and Savitzky-Golay smoothing. The resulting preprocessed spectra are illustrated in Fig. 7.

Following complete preprocessing, the spectral data exhibited marked improvement, yielding smooth, well-defined baselines (~ 0.5–2 × 10^−3^ normalized intensity units) with minimal noise fluctuations for both samples. As shown in Fig. 7, the area normalization (“Spectral preprocessing pipeline” section, Step 4) successfully preserved the intrinsic spectral shape and relative peak heights, ensuring that the ~ 1.5 × intensity difference between the samples reflects genuine chemical variations rather than processing artifacts. Consequently, the dominant EHMC peak in the sunscreen-contaminated sample (blue line) is sharply resolved at 480–520 nm with well-defined shoulders, clearly elevating above the baseline throughout the 480–700 nm region. Furthermore, secondary features at ~ 550–600 nm and weak contributions at 700–800 nm are now clearly visible as distinct spectroscopic signals rather than noise spikes. In contrast, the uncontaminated salt sample (green line) maintains a smooth baseline with only subtle spectral structure (~ 0.5–1.0 × 10^−3^). Ultimately, these highly refined spectral outcomes serve as the direct input for Sparse PCA (“Sparse principal component analysis” section) and SVM classification (“Support vector machine classification performance” section), making this rigorous preprocessing quality directly responsible for the robust classification accuracy achieved in subsequent analyses.

### Hyperparameter optimization

#### Selection of number of sparse principal components

Systematic cross-validation was used to identify the ideal number of components (*k*). The eigenvalue spectrum and variance explained by each component were first calculated using standard PCA. After that, *k* was changed from 1 to 12, and the first *k* components were used to train a SVM classifier. Five-fold cross-validation was used to assess performance. The findings showed that:

- *Performance plateau* Classification accuracy reached an optimal plateau between components 4 and 5, demonstrating excellent consistency across validation trials.
- *Variance threshold* The first 4 components collectively explained 81.16 percent of the cumulative spectral variance.
- *Diminishing returns* While component 5 maintained high accuracy, its marginal contribution to performance was negligible and did not justify the added dimensionality. Beyond component 5, accuracy began to decline, presenting a clear overfitting signature.

**Fig. 8:**
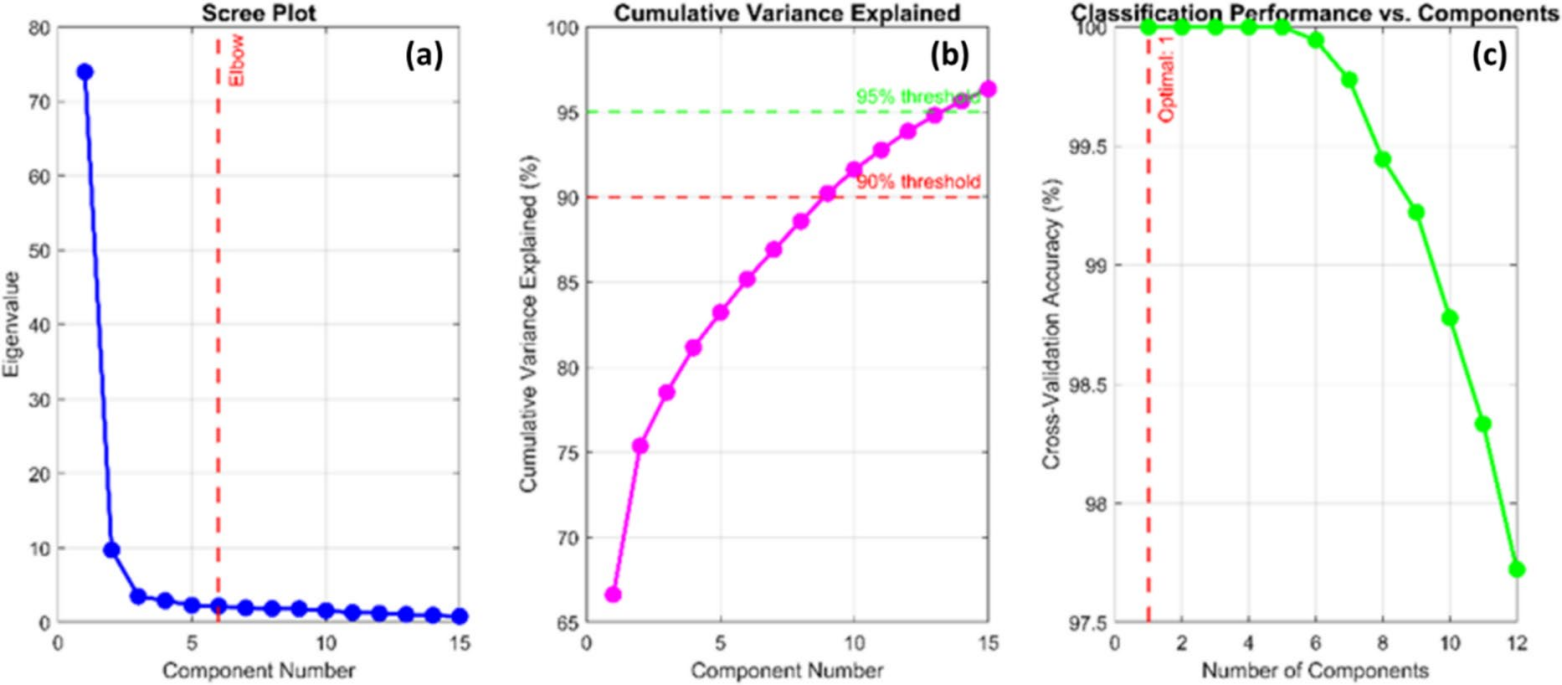
Diagnostic evaluation of sparse PCA component selection: **a** scree plot of eigenvalues (left), b cumulative variance explained (center), and **c** classification performance versus components (right). The convergence of these metrics supports k = 4 as the optimal number of components for downstream classification

Following this,* k* = 4 components were selected as the optimal threshold. By applying the principle of model parsimony, *k* = 4 maximizes discriminatory power while utilizing the simplest possible architecture, thereby reducing computational complexity and the risk of overfitting, as depicted in Fig. 8.

As shown in Fig. 8, three diagnostic analyses used to evaluate the adequacy of the selected number of sparse PCA components. The Scree Plot (Fig. 8a) displays the eigenvalues in descending order, showing a distinct elbow at component 6, where subsequent components contribute only marginal variance. The Cumulative Variance Explained plot (Fig. 8b) indicates that four components capture 81.16% of the total spectral variance, approaching 95% by approximately nine components. Meanwhile, the Classification Performance vs. Components plot (Fig. 8c) shows that accuracy stabilizes at component 4, with the fifth component providing negligible improvement and an increased likelihood of overfitting. These results collectively confirm that selecting k = 4 provides an optimal balance between dimensionality reduction and classification reliability.

#### Sparsity parameter (${\boldsymbol{\lambda}}$) optimization

**Fig. 9:**
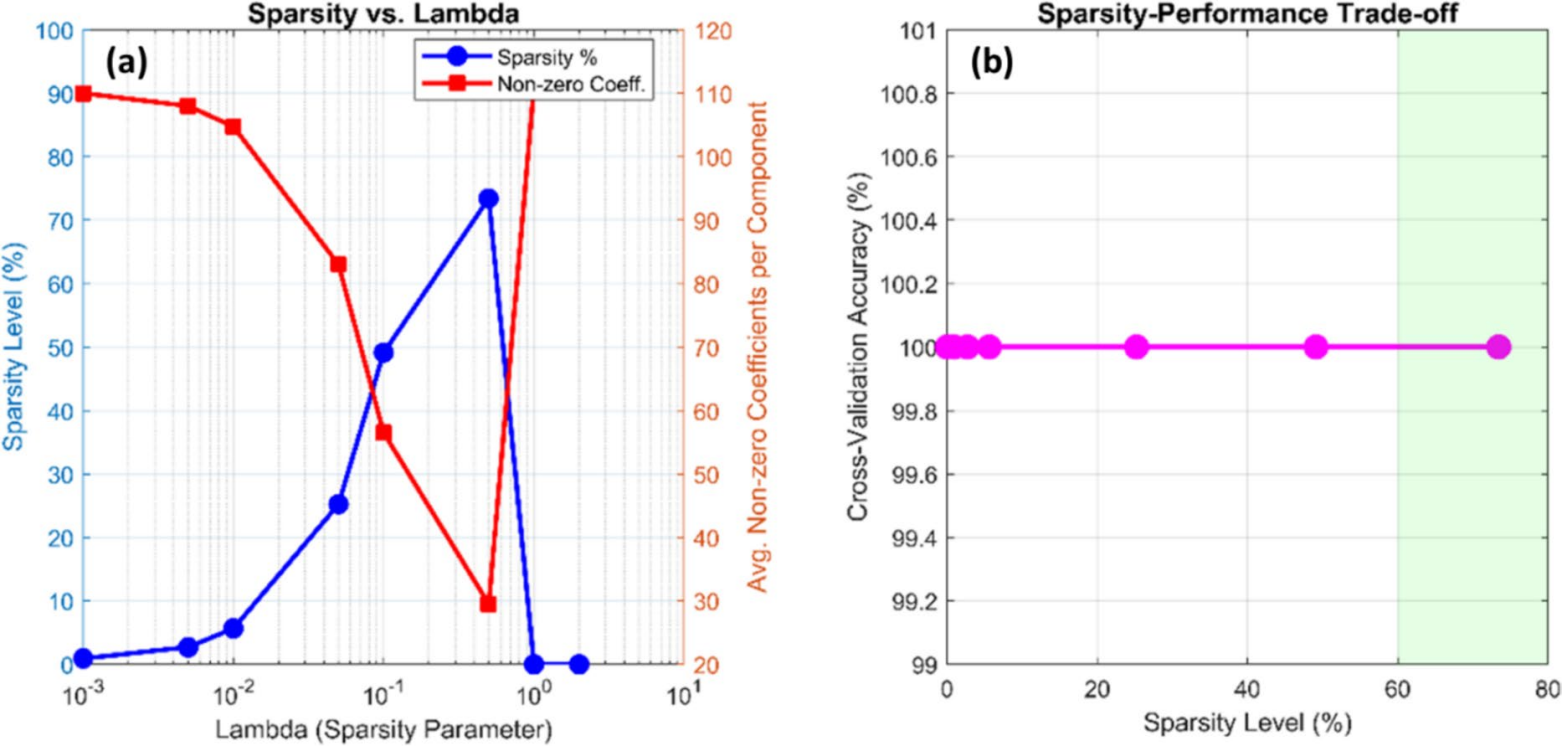
Sparsity–performance trade-off in sparse PCA parameter optimization. **a** Sparsity versus lambda: the blue curve shows the sparsity percentage, and the red curve indicates the average number of non-zero coefficients per component. As λ increases from 10^−3^ to 10^0^, sparsity rises to ~ 73%, while non-zero coefficients drop from ~ 110 to ~ 30. **b** Sparsity–performance trade-off: cross-validation accuracy remains nearly constant across 0–80% sparsity, with only minor degradation at extreme regularization levels. The optimal λ = 0.5 (73.4% sparsity) achieves maximal interpretability with no loss in classification accuracy

Grid search was used to maximize the sparsity parameter λ, which controls the degree of L₁ regularization: λ ∈ {0.001, 0.005, 0.010, 0.050, 0.100, 0.500, 1.000, 2.000}. Sparse PCA was calculated for each λ value, and then SVM training and performance evaluation using fivefold cross-validation were performed. Figure 9 presents the diagnostic results of this optimization, illustrating how varying λ values influence both the sparsity of loading vectors and the overall classification performance of the sparse PCA–SVM pipeline. The analysis explores λ values ranging from 10^−3^ to 10^0^, highlighting the transition from dense representations toward highly sparse, feature-selective models.

As shown in Fig. 9, increasing λ caused a progressive rise in sparsity from nearly 0% to approximately 73%, while the average number of non-zero coefficients per component decreased from around 110 to 30 (Fig. 9a). Concurrently, classification accuracy remained consistently high across most of this sparsity range (Fig. 9b). The optimal configuration was obtained at λ = 0.5, which yielded 73.4% sparsity while maintaining robust classification performance. Under this condition, only 118 non-zero coefficients out of 444 total were retained, corresponding to a 73.4% reduction in dimensionality. Additionally, components 2–4 exhibited extreme sparsity (93.7–94.6%), each retaining only 6–7 non-zero loadings concentrated in the 480–600 nm spectral region, corresponding to the dominant EHMC fluorescence bands. These results demonstrate that the model effectively isolates the most discriminative spectral features while eliminating redundant information, producing interpretable and computationally efficient component representations.

### Sparse principal component analysis

Sparse PCA was utilized as the principal method for dimensionality reduction and feature selection. Sparse PCA, in contrast to traditional PCA that emphasizes variance retention and generates dense loading vectors encompassing all original variables, employs an explicit L₁ (Lasso) regularization penalty that promotes sparsity in loading vectors, resulting in several coefficients being precisely zero. With optimal parameters (k = 4 components, λ = 0.5), Sparse PCA was applied to the preprocessed, standardized spectral dataset. This process accomplished two critical objectives:

1. *Chemical feature identification* Identification of the chemically important wavelength areas linked to sunscreen fluorescence emission.
2. *Noise suppression* Mitigation of noise and extraneous spectral data, enhancing feature quality for subsequent classification.

**Fig. 10:**
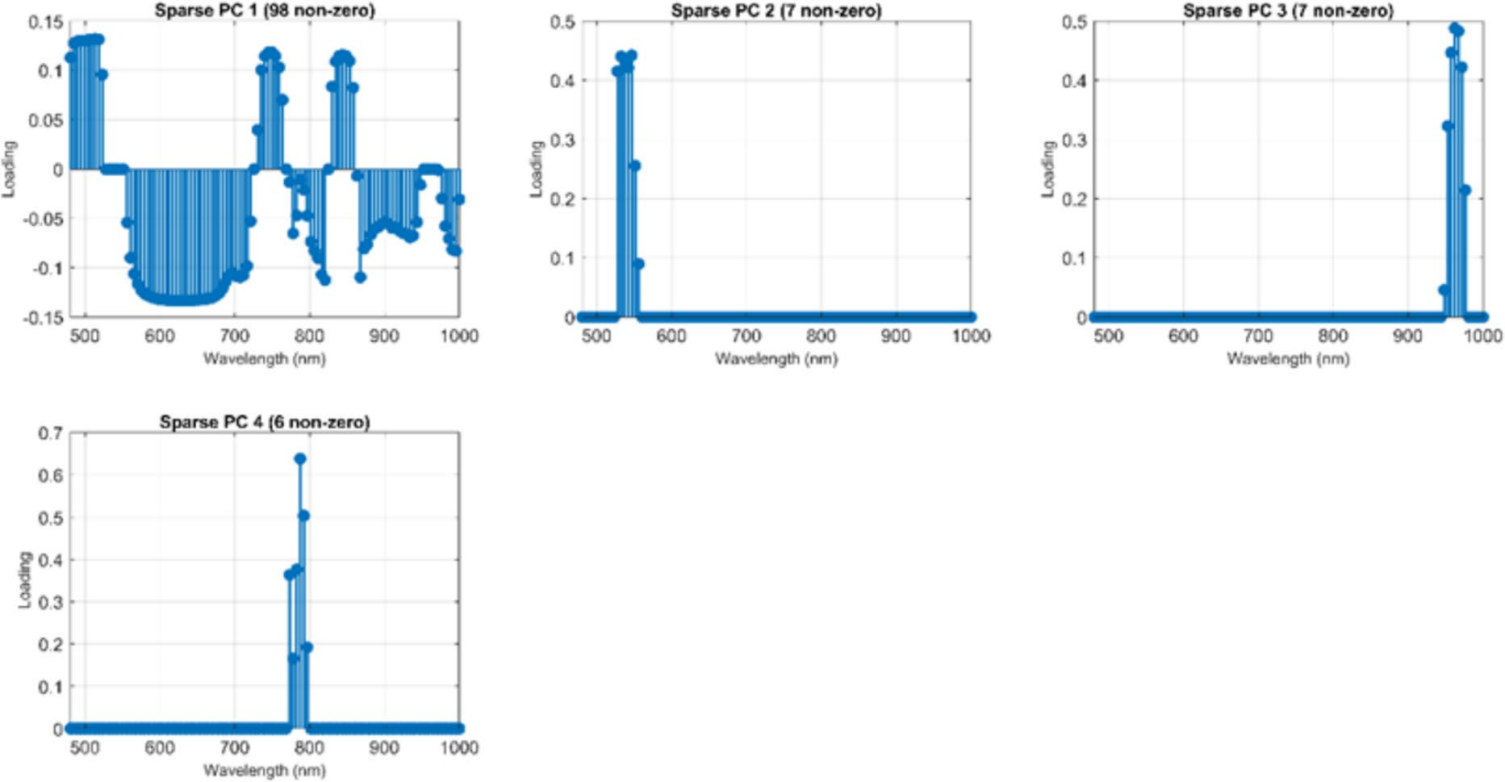
Sparse PCA loading distributions for four principal components. Non-zero loadings are shown across the 500–1000 nm spectral range. PC 1 (98 non-zero) spans the spectrum broadly. PC 2 (7 non-zero) focuses on 480–600 nm, matching EHMC steady-state fluorescence. PC 3 (7 non-zero) targets 980–1000 nm, capturing far-red photoinduced absorption from photodegradation radicals. PC 4 (6 non-zero) selects intermediate wavelengths (~ 800 nm), corresponding to transient absorption. The complementary wavelength selectivity across PCs 2–4 enables robust sunscreen detection using multiple spectral features

The sparse loading vectors derived from Sparse PCA revealed that components 2–4 predominantly contained non-zero loadings within the 480–600 nm range, corresponding to the characteristic extended-conjugated absorption and fluorescence bands of EHMC, the primary UV filter in the sunscreen formulation. Figure 10 illustrates the four sparse principal components identified, highlighting the distribution of non-zero loadings across the 500–1000 nm spectral window. Sparse PC 1, with 98 non-zero loadings, shows a broad distribution spanning the full spectral range, whereas components 2–4 exhibit highly selective loadings in specific regions, indicating targeted spectral sensitivity for distinct chemical signatures.

As shown in Fig. 10, the physical relevance of the Sparse PCA–selected bands can be directly interpreted in terms of the known photophysical behavior of organic sunscreen molecules and their photoproducts. Sparse PCA does not arbitrarily select wavelengths; instead, it isolates narrow spectral regions that maximize variance associated with specific radiative and non-radiative processes. Specifically, Sparse PC 2 (480–600 nm) aligns with the steady-state fluorescence emission of EHMC, which arises from radiative relaxation of photoexcited π–π* electronic states following blue excitation. This visible fluorescence band has been widely reported for cinnamate-based UV filters and serves as a direct optical fingerprint of intact sunscreen residues. In contrast, Sparse PC 3 (980–1000 nm) captures weak but distinctive far-red to NIR photoinduced absorption, which is attributed to long-lived photodegradation radicals and triplet-state absorption formed during EHMC photolysis. These non-fluorescent species do not emit strongly but introduce absorption features detectable in the NIR, providing complementary evidence of contaminant presence. Finally, Sparse PC 4, with 6 non-zero loadings, targets intermediate wavelengths around 800 nm, corresponding to transient absorption pathways, likely associated with excited-state relaxation and intermediate photochemical products. Although weaker than steady-state fluorescence, these bands contribute additional discriminatory power by encoding secondary photophysical dynamics. The orthogonal wavelength selectivity of components 2–4 demonstrates that sunscreen detection is governed by complementary photophysical mechanisms rather than a single fluorescence peak. This multi-mechanism spectral representation enhances classification robustness, ensuring reliable discrimination even under temporal variations in excited-state populations caused by differences in illumination history and photochemical aging, thereby reinforcing both the interpretability and stability of the Sparse PCA framework.

### Support vector machine classification performance

#### Class separation in feature space

A SVM with RBF kernel was trained on the sparse PCA-transformed features to differentiate between areas with clean salt and those contaminated with sunscreen. SVM configuration specification were:

- *Kernel* RBF with kernel scale auto-optimized.
- *Box constraint (C)* 1.0 (regularization parameter).
- *Class labels* 0 = clean salt, 1 = sunscreen-contaminated.
- *Standardization* Enable (zero-mean, unit-variance normalization).

**Fig. 11:**
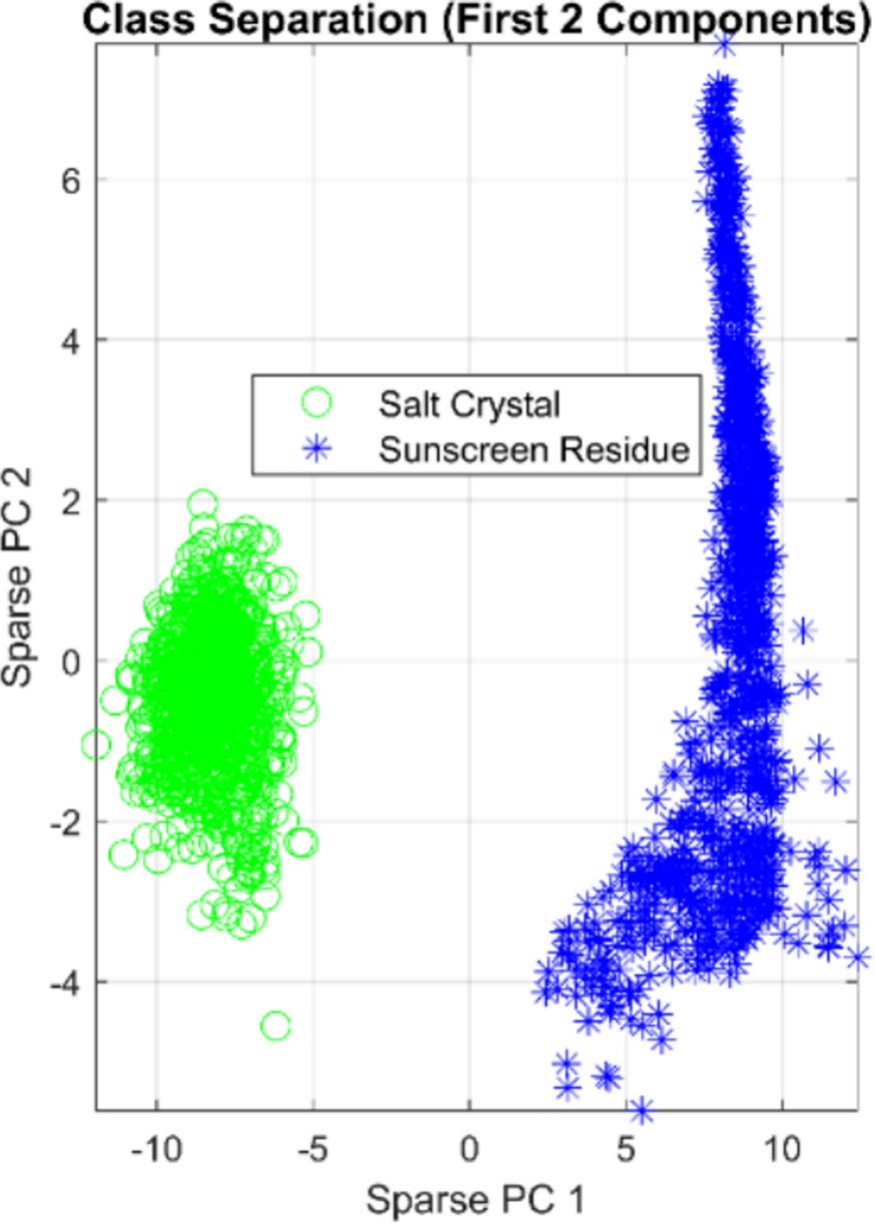
Scatter plot of Sparse PC 1 versus Sparse PC 2 illustrating class separation. Clean salt pixels (green circles) cluster in the lower-left quadrant, while sunscreen-contaminated pixels (blue asterisks) cluster in the upper-right quadrant

Through soft-margin formulation, the SVM determines the hyperplane that simultaneously reduces classification errors while maximizing the margin between class centroids. By using kernel techniques to create suitable nonlinear decision limits in high-dimensional feature space, SVMs accomplish robust classification even in cases where spectral overlaps result in deceptive training data. The SVM’s theoretical underpinnings maximize the margin between classes, which offers strong generalization guarantees. This trait is especially useful when training sample quantities are constrained, as is frequently the case in specialized analytical applications. Figure 11 presents the scatter plot of Sparse PC 1 versus Sparse PC 2 for all training samples, clearly illustrating the complete spatial separation between clean salt pixels and sunscreen-contaminated pixels in the transformed feature space.

As shown in Fig. 11, Clean salt pixels (green circles) cluster in the lower-left quadrant (negative PC 1, near-zero PC 2), while sunscreen-contaminated pixels (blue asterisks) occupy the upper-right quadrant (positive PC 1, positive PC 2), forming a dense, well-defined cluster. The complete spatial separation between the two classes demonstrates minimal spectral overlap and confirms that the Sparse PCA transformation successfully linearizes the class boundary. This orthogonal arrangement provides a clear basis for SVM classification and accounts for the high accuracy and robustness observed across all validation protocols.

#### Cross-validation performance

To rigorously evaluate model stability and generalizability, tenfold stratified cross-validation was performed on the 1800-pixel labeled dataset, comprising 900 clean and 900 sunscreen-contaminated pixels. The analysis demonstrated that the sparse-PCA features and SVM classifier achieved nearly perfect results across all folds (accuracy ≈ 100%, with precision, recall, and F1-score ≈ 1.0). This behavior aligns with the pronounced fluorescence contrast observed between the two classes and indicates an upper limit on attainable performance under optimal, precisely controlled conditions. Consequently, to obtain a more realistic estimate of the model’s behavior on heterogeneous scenes, we subsequently evaluated it against independent ground-truth annotations at both spectral and pixel-wise spatial levels (“Ground truth-based validation” section).

#### Full image pixel-wise classification

**Fig. 12:**
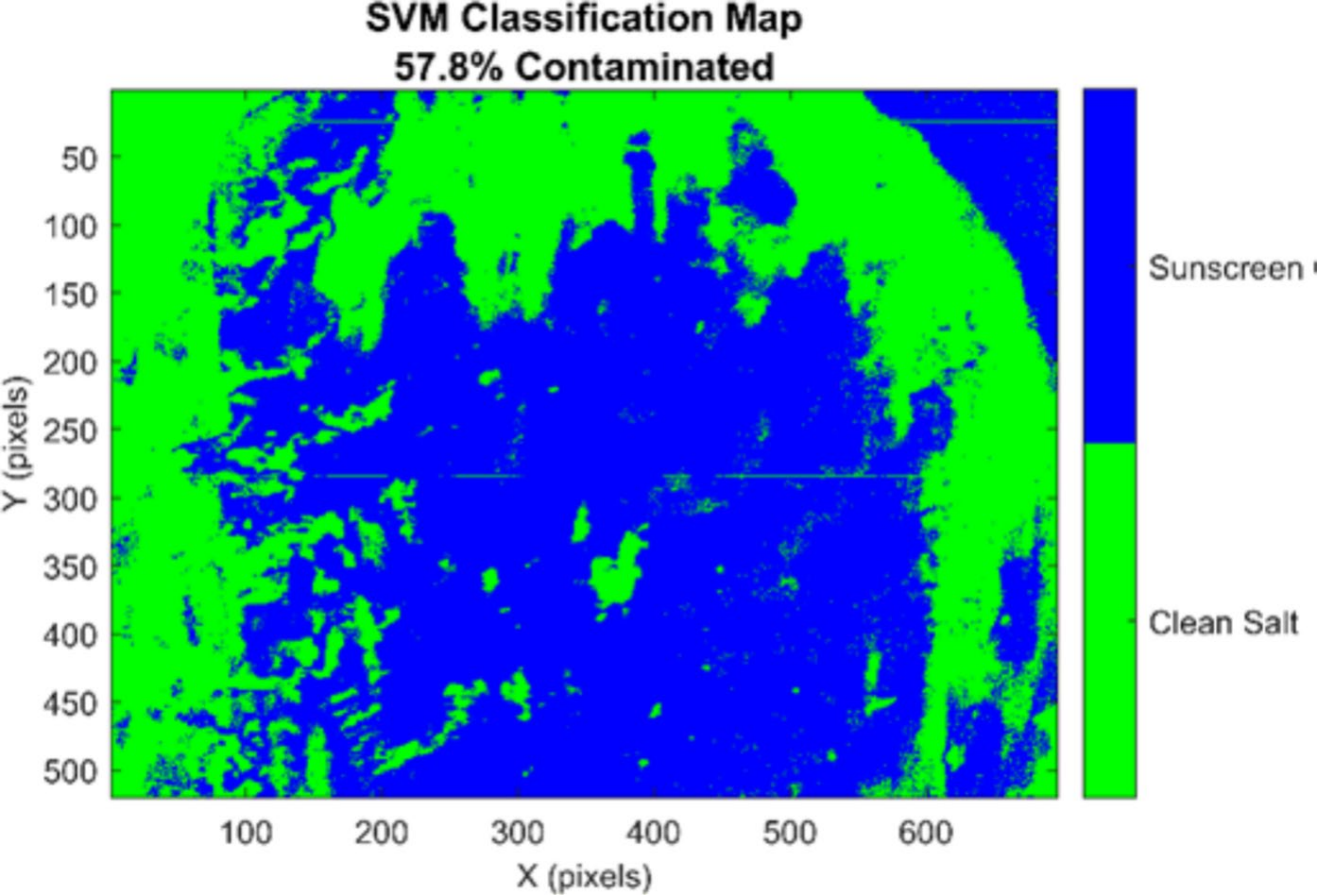
SVM classification map showing contamination distribution. Complete-image SVM classification result with identical spatial dimensions to the original HS acquisition. Pixels are colored based on predictions: green indicates clean salt (~ 42.2% of image), while blue indicates sunscreen contamination (~ 57.8%). The classification reveals spatially clustered contamination patterns, with sunscreen residues appearing in discrete regions distributed across the salt surface rather than uniform coverage. This spatial clustering is consistent with heterogeneous crystallization, where sunscreen components preferentially accumulate in specific zones due to differential evaporation rates and crystal plane interactions

The trained SVM classifier was applied to the entire hyperspectral image, comprising 361,920 pixels (520 rows × 696 columns), as illustrated in Fig. 12. Pixel-wise SVM predictions are color-coded: green = uncontaminated salt (class 0), blue = sunscreen-contaminated salt (class 1).

The key observations in Fig. 12 is as follows:

- *Heterogeneous contamination distribution* 57.8% pixels classified as contaminated, 42.2% clean.
- *Coherent spatial clustering* Contaminated regions form continuous clusters in image center and lower portions, consistent with physical expectations for uneven crystallization-based contamination.
- *No scattered misclassifications* Absence of scattered noise artifacts indicates robust, spatially-consistent predictions.
- *Clear boundary definition* Sharp transitions between contaminated and clean regions reflect actual physical salt crystal structure.

#### Ground truth-based validation

**Fig. 13:**
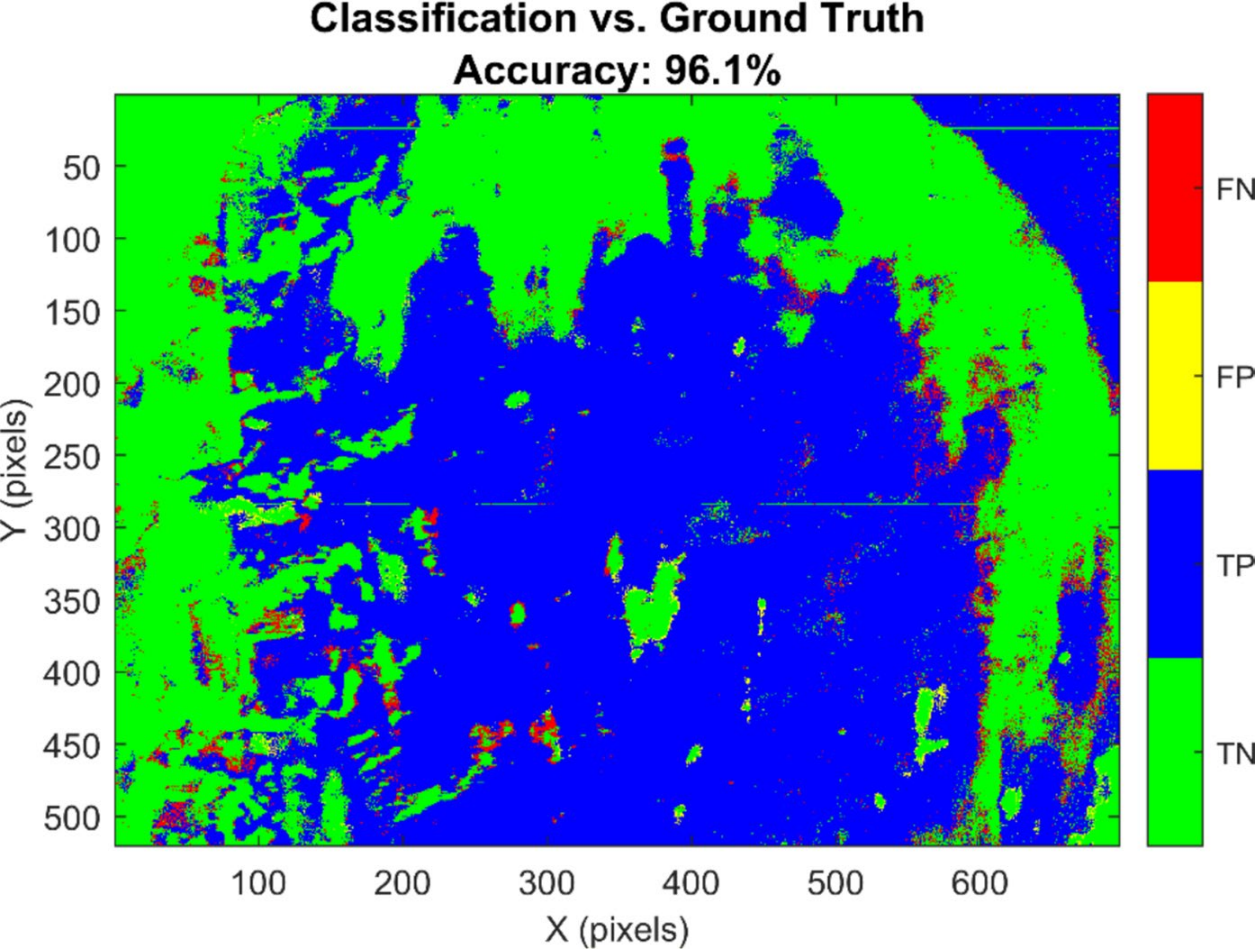
Spatial error map comparing the SVM classification results against the ground truth reference the map visualizes pixel wise classification performance blue regions represent true positives (correctly detected sunscreen), green regions represent true negatives (correctly identified clean salt), yellow regions indicate False Positives (over-detection), and red regions indicate false negatives (missed contamination). The visual dominance of blue and green pixels confirms the model’s high spatial fidelity, achieving an overall accuracy of 96.1% with minimal scattered misclassifications

In addition to ROI-based internal validation, the classifier was evaluated against the independent ground truth described in “Ground truth extraction” section. First, we considered the balanced GT spectral validation set of 4000 pixels (2000 salt and 2000 contaminated) randomly sampled from the GT mask. On this dataset, the model achieved an accuracy of 96.65%, with precision 0.9854, recall 0.9470, and F1-score 0.9658. These ground-truth–based metrics confirm that the high discriminative power observed on ROI spectra largely transfers to independent data, while providing a more conservative and realistic estimate of performance than ROI-based cross-validation alone. Second, a full pixel-wise comparison between the SVM classification map and the complete GT mask was performed over all labeled pixels in the HS image as shown in Fig. 13. This analysis yielded 207,068 true positives, 140,714 true negatives, 2120 false positives, and 12,018 false negatives, corresponding to an overall spatial accuracy of 96.09%, with precision 0.9899, recall 0.9451, and F1-score 0.9670. Misclassifications were predominantly located along the interfaces between contaminated and clean crystals, whereas large homogeneous regions were almost perfectly labeled. These ground-truth–based results provide a realistic assessment of the method’s performance for practical contamination mapping and are therefore used as the primary indicators of effectiveness in this study.

#### Independent sample detection

**Fig. 14:**
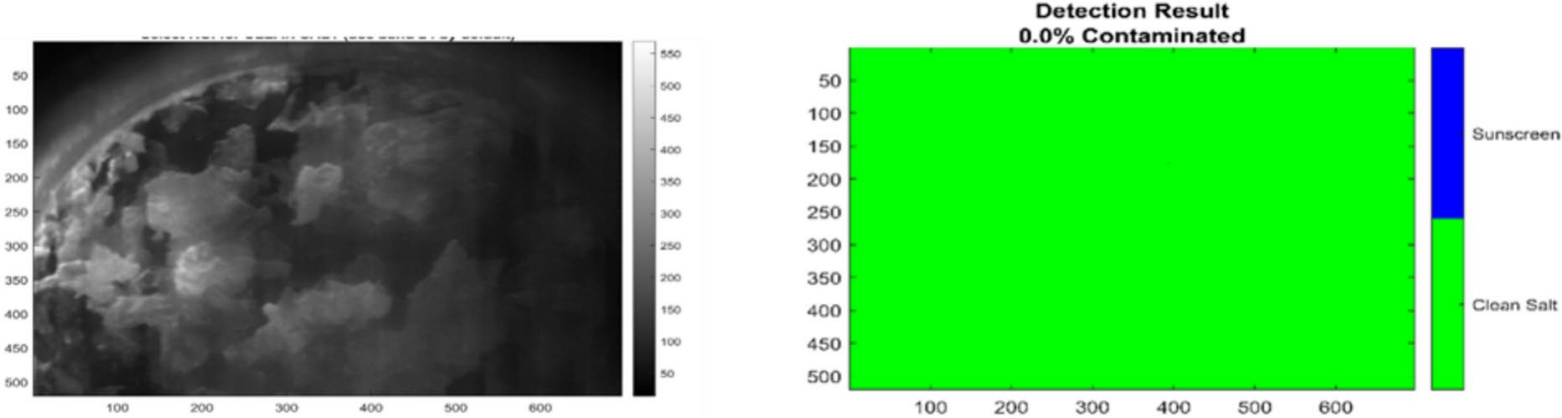
Detection results on an independent, uncontaminated salt sample (different crystallization batch and acquisition time). Left panel: HS image (band 23). Right panel: SVM classification result, with all pixels correctly classified as clean salt (green coloration). The results confirm robust model generalization and absence of false positives on independent data

To evaluate the generalizability of the trained Sparse PCA + SVM model, classification was performed on an independently prepared salt sample produced using the same protocol as the training set but from a different crystallization batch and acquired at a different time. Figure 14 presents the HS image of this new sample alongside the corresponding SVM classification results, allowing assessment of the model’s performance on truly independent data.

As shown in Fig. 14, The SVM classification results demonstrate complete recognition of the sample as uncontaminated, with all 361,920 pixels assigned to the clean salt class and no false positives detected. This outcome confirms that the model reliably generalizes to previously unseen data, indicating that the learned classification boundaries capture genuine, reproducible spectroscopic differences between clean and contaminated salt. Independent verification of the sample confirmed the absence of sunscreen residues, further validating the robustness, accuracy, and practical applicability of the approach for real-world monitoring of food-grade salt.

**Fig. 15:**
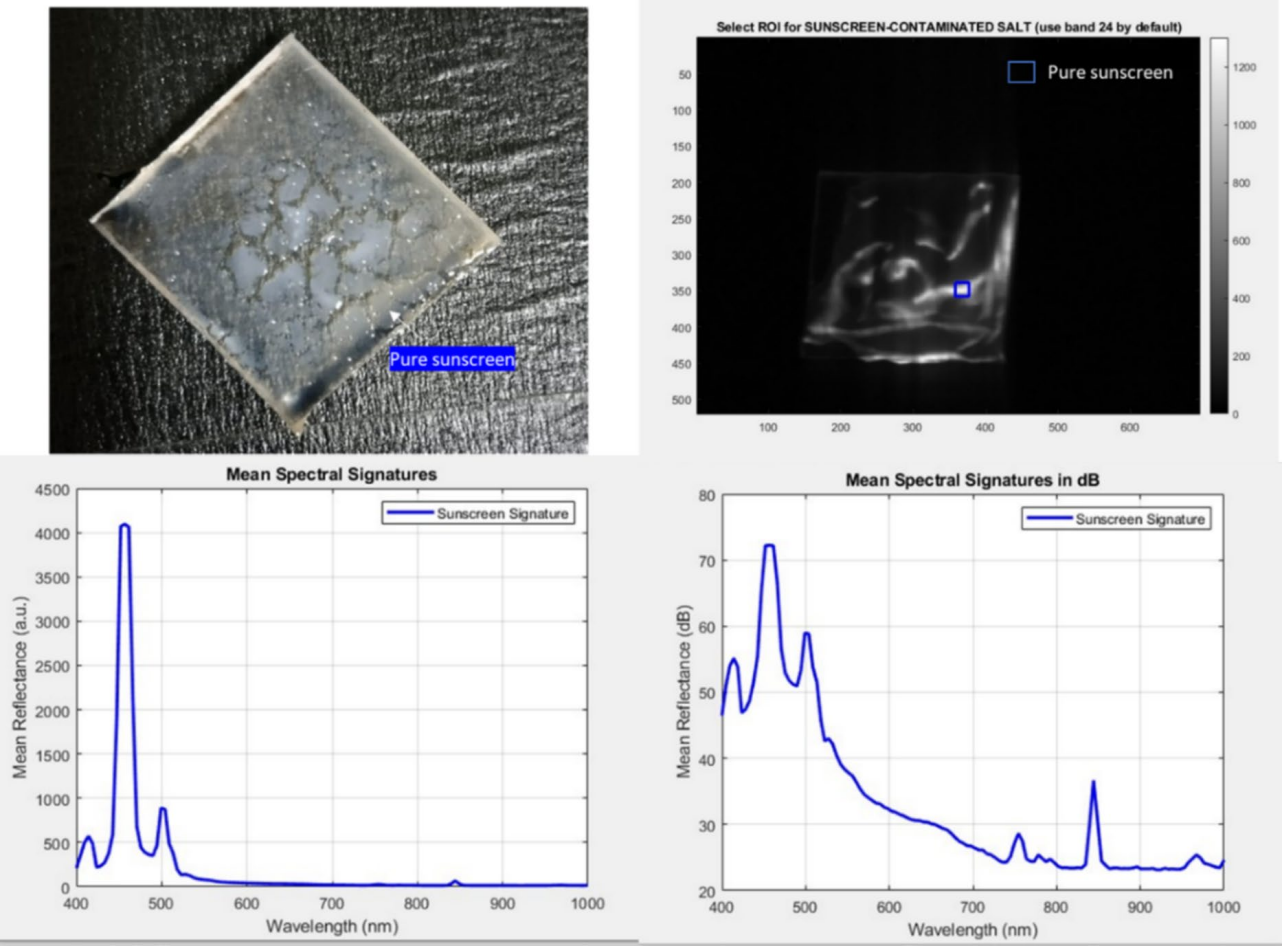
Experimental validation of the pure sunscreen spectral signature. (Top Left) Visual image of the pure sunscreen reference sample applied to a glass slide. (Top Right) Hyperspectral fluorescence image (band 23, ~ 512 nm) showing the ROI selection for the pure sunscreen film. (Bottom Left) Mean spectral signature in linear scale, displaying a distinct fluorescence emission maximum at 480–520 nm. (Bottom Right) Logarithmic (dB) scale representation of the emission spectrum. The identical spectral profile observed here and in the contaminated salt samples (Fig. 5) confirms that the detection is driven by the intrinsic molecular fluorescence of the sunscreen UV filters

To unequivocally attribute the detected contamination on the salt crystals to the sunscreen itself rather than to environmental or marine interferents, a controlled reference experiment was conducted using the sunscreen formulation alone. A pure layer of the sunscreen was deposited on a glass slide. Hyperspectral LIF measurements acquired from this dried sunscreen residue exhibited a spectral signature that was highly consistent with the fluorescence features extracted from the residue observed on the salt crystal surfaces. In particular, the dominant steady-state emission band and the long-lived photoinduced absorption components identified in the isolated sunscreen residue (Fig. 15) showed strong correspondence with the contaminated salt samples (Fig. 5 and Fig. 6). This spectral congruence confirms that the fluorescence response detected on the salt crystals originates from persistent sunscreen-derived chromophores rather than from transient excitation artifacts or background marine organic matter. When considered alongside the zero false-positive rate observed for the clean salt control group (Fig. 14), these results collectively demonstrate that the Sparse PCA–SVM framework is selectively responsive to the intrinsic photophysical signature of the sunscreen residue, even after evaporation and crystallization, thereby providing robust chemical specificity under realistic environmental conditions.

#### Limit of detection (LOD)

**Fig. 16:**
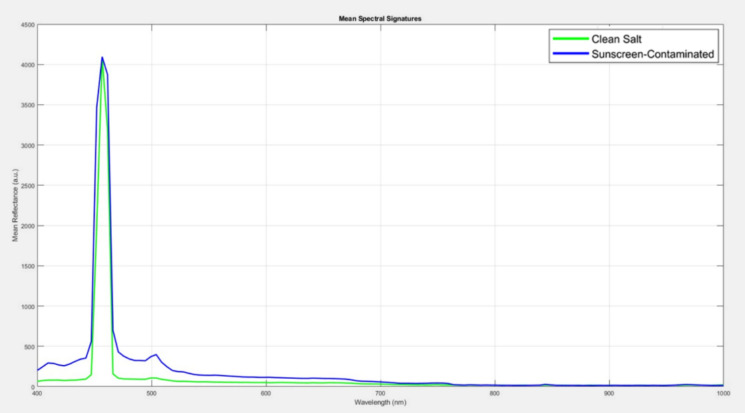
Average raw LIF intensity spectra for clean salt crystals and trace sunscreen residues at a 5 mg/L concentration under 450 nm blue laser excitation

**Fig. 17:**
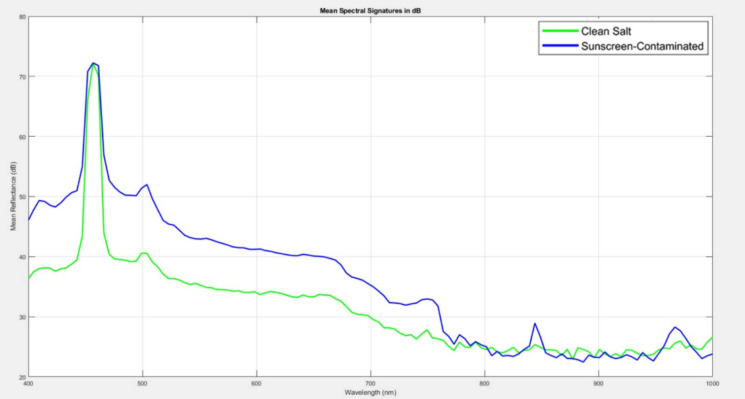
Average normalized LIF intensity spectra expressed in decibels (dB) for clean salt crystals and trace sunscreen residues (5 mg/L) under 450 nm blue laser excitation

To rigorously evaluate the analytical sensitivity of the proposed framework, preliminary screening was conducted across a trace concentration gradient. For the primary classification pipeline discussed in the preceding sections, the 10 mg/L concentration was deliberately selected as a highly challenging case study. At this trace level, the sunscreen residues are visually imperceptible and heavily obscured by the complex optical scattering of the crystalline salt matrix. This provided an ideal, rigorous scenario to prove the outstanding spatial mapping capabilities and classification accuracy of the proposed Sparse PCA–SVM architecture. To further assure the operational limits of the LIF-HSI approach, validation was extended to the lower concentration thresholds. Specifically, the exact experimental protocol was repeated to evaluate a 5 mg/L sample. Following the previously established methodology, food-grade sea salt was uniformly doped with the 5 mg/L sunscreen solution, dried under controlled conditions to evaporate the solvent, and subsequently scanned using the identical LIF-HSI configuration (continuous-wave 450 nm blue laser excitation at a 40 cm working distance). Figure 16 Average raw LIF intensity spectra for clean salt crystals and trace sunscreen residues at a 5 mg/L concentration under 450 nm blue laser excitation. illustrates the average raw LIF intensity curves for both the clean salt crystals and the 5 mg/L sunscreen residues. While, Fig. 17 presents the average normalized LIF intensity curves expressed in decibels (dB) for this concentration.

As shown in Fig. 16, the resulting spectral data directly illustrates the optical limitations encountered at this lower concentration (5 mg/L). At this trace level, the inherent fluorescence emission of the sunscreen is severely diminished, and the absolute signal intensity drops significantly, nearly merging with the baseline optical scattering signature of the clean salt crystals. While the logarithmic scaling in Fig. 17 amplifies the relative emission strengths, it mathematically confirms that the spectral margin between the clean and contaminated samples at 5 mg/L becomes critically narrow. At this proximity, the optical signal-to-noise ratio degrades to a point where the inherent physical heterogeneity and surface scattering of the salt matrix completely overshadow the chemical fluorescence fingerprint. Consequently, attempting to apply the classification pipeline below 10 mg/L would result in overlapping decision boundaries and an unacceptable increase in false positive classifications due to background noise. Therefore, this empirical spectral convergence confirms that 10 mg/L is the practical LOD for this specific LIF-HSI hardware configuration, successfully justifying its use as the optimal benchmark for evaluating the proposed framework.

### Comparative analysis: sparse PCA versus standard PCA

To quantify the advantages of the proposed sparse modeling approach, a baseline comparative study was conducted using standard PCA without regularization. The baseline model utilized the same number of components (*k* = 4) and SVM classifier parameters as the proposed framework to ensure a fair comparison.

#### Interpretability of spectral features

**Fig. 18:**
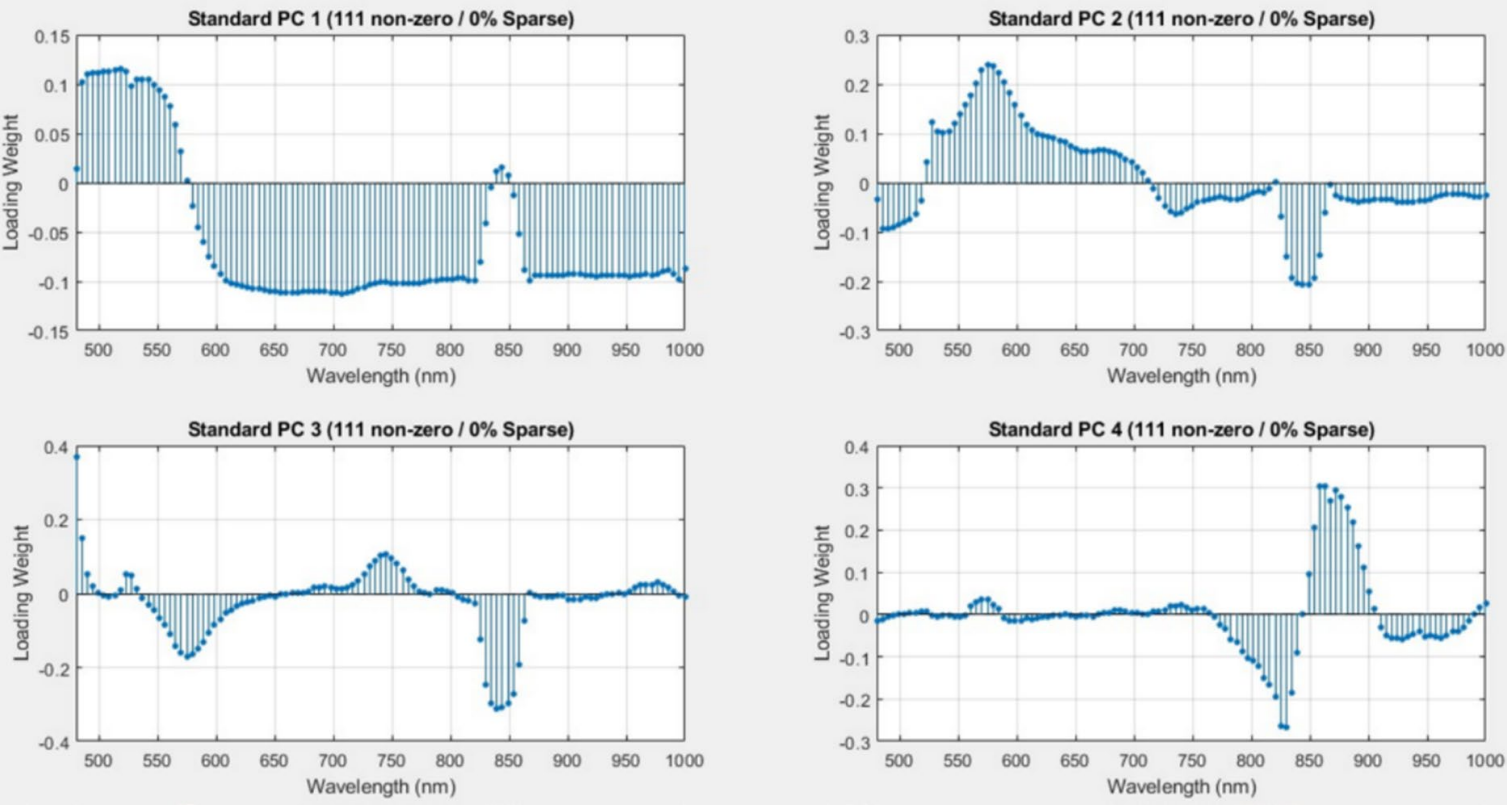
Standard PCA loading vectors for the first four principal components. Unlike the sparse features derived in this study, standard PCA produces dense loadings with non-zero weights distributed across all 111 spectral bands. This lack of wavelength selectivity incorporates background noise into the feature space, reducing the chemical interpretability of the model

A fundamental limitation of standard PCA in high-dimensional spectral analysis is the generation of ‘dense’ loading vectors. As illustrated in Fig. 18, the standard PCA loadings exhibit non-zero coefficients across the entire 480–1000 nm range. This indicates that the principal components are linear combinations of all spectral bands, effectively mixing diagnostic sunscreen signals with background noise and scattering artifacts. In contrast, the Sparse PCA loadings (previously shown in Fig. 10) are zero-sparse, isolating only the physically relevant fluorescence peaks (e.g., 480–600 nm) while suppressing non-informative wavelengths.

#### Classification performance benchmarking

**Fig. 19:**
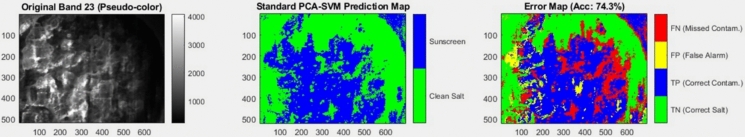
Comparative classification performance of the baseline standard PCA–SVM model. (Left) Pseudo-color representation of the raw HS band 23. (Center) The prediction map generated using standard PCA features, showing noisy and inconsistent classification. (Right) The spatial error map reveals a high rate of misclassification (Accuracy: 74.3%), with extensive False Negatives (red) and False Positives (yellow), demonstrating that standard PCA features fail to capture the subtle contrast required for robust contamination mapping

The impact of this feature quality on classification was evaluated by training the SVM on the standard PCA scores. The resulting performance map, shown in Fig. 19, reveals significant degradation in spatial accuracy compared to the proposed method. The Standard PCA–SVM baseline achieved a global pixel-wise accuracy of only 74.3%, characterized by substantial false negatives (red regions) in areas of lower fluorescence intensity and false positives (yellow regions) driven by background noise. Conversely, the Sparse PCA–SVM pipeline achieved 96.1% accuracy. This 21.8% performance gap confirms that the L_1_ regularization is not merely a mathematical abstraction but a critical requirement for robust detection in noisy, HS environments.

## Discussion

From a food safety and environmental perspective, these findings highlight the potential of LIF-HSI as a non-destructive screening tool for organic contaminants in sea salt and, by extension, in other crystalline or particulate food matrices. Unlike conventional chromatographic methods, which are destructive, time-consuming, and require extensive sample preparation, the proposed optical–computational workflow provides rapid, spatially resolved information on contaminant distribution. This capability is particularly relevant for monitoring products originating from coastal regions with high recreational or industrial activity, where sunscreen-derived UV filters and other organic pollutants are likely to accumulate.

While GC-MS/LC-MS remains the gold standard for analytical quantification, offering detection limits in the nanogram per liter (ppt) range and wide linear dynamic ranges, these figures of merit come at the cost of sample destruction and zero spatial resolution. In contrast, the proposed LIF-HSI framework operates as a qualitative screening tool. By validating the method at a concentration of 10 mg/L, we demonstrate its capability to detect contamination levels relevant to environmental accumulation without sample preparation. A comparison of key operational figures of merit is presented below:

- *Detection capability* GC-MS quantifies trace residues (ppt–ppb levels), whereas the current LIF-HSI model is optimized for rapid classification at screening thresholds (ppm range), sufficient for identifying gross contamination in industrial quality control.
- *Spatial resolution* GC-MS provides a single bulk concentration value per sample. LIF-HSI offers a spatial resolution of about 40 µm /pixel (based on the SOC710 sensor geometry), enabling the identification of localized contamination ‘hotspots’ on crystal surfaces that bulk analysis might average out.
- *Throughput* The LIF-HSI workflow allows for real-time acquisition (seconds per scan) compared to the 4–8 h workflow required for chromatographic extraction and analysis.

It is critical to distinguish the detection mechanism of the proposed framework from standard reflectance-based HS imaging. In passive reflectance, the limit of detection is constrained by the linear degradation of contrast between the contaminant and the background as concentration decreases. In contrast, the proposed LIF methodology employed here leverages an active “signal-against-darkness” principle (Wu & Barner-Kowollik, 2023).

The 450 nm excitation source exploits the high quantum yield of the sunscreen’s cinnamate chromophore, generating a distinct Stokes-shifted emission peak at 480–600 nm. Because the crystalline salt matrix exhibits negligible fluorescence in this window, the background noise is effectively suppressed, maximizing the signal-to-noise ratio. Consequently, the detection capability is not defined by a scalar intensity threshold, but instead relies on recognition of the intrinsic chemical print, namely the specific spectral shape of the fluorophore, which remains spectrally invariant across concentration gradients. This characteristic enables the Sparse PCA algorithm to isolate the contaminant’s unique signature even when its spatial footprint is minimal, providing a sensitivity advantage that passive optical gradient methods cannot replicate.

Nevertheless, several limitations and opportunities for future work should be noted. First, while this study utilized seawater from a single primary coastal region, the analytical robustness was verified by cross-referencing the detected contamination signal against the spectral signature of pure commercial sunscreen (Fig. 15). Consequently, the detection capability is driven by the contaminant’s specific chemical identity rather than the background seawater matrix, suggesting the method remains effective across varying levels of salinity or microbial composition found in other marine areas. Second, the ground truth relies on manual annotation of fluorescence images, which, although carefully performed, may introduce subjective bias, especially at boundaries and in low-signal regions. Semi-automated or consensus-based labeling strategies could strengthen future ground-truth datasets. Third, the experiments were conducted under controlled laboratory conditions with a fixed excitation wavelength; exploring multi-wavelength excitation or alternative illumination geometries could further improve sensitivity to a wider range of sunscreen filters and related organic contaminants. Finally, because the LIF-HSI data cubes generated in this study represent a novel, proprietary dataset uniquely developed by our research group, future work will involve a dedicated empirical study to comprehensively benchmark the performance, computational efficiency, and spatial mapping robustness of various machine learning and deep learning architectures against this specific matrix.

Based on the promising outcomes presented in this study, the integration of the LIF method into online quality control systems at salt processing or packaging facilities could enable real‑time monitoring. The computational efficiency of the Sparse PCA–SVM pipeline makes it particularly well-suited for processing streaming HS data on high-speed production lines.

## Conclusion

This study established a robust analytical framework integrating Laser-Induced Fluorescence (LIF) Hyperspectral Imaging with Sparse PCA–SVM analysis for the rapid, non-destructive detection of sunscreen contaminants in sea salt. By targeting the characteristic fluorescence emission of UV filters under 450 nm excitation, the proposed model achieved a pixel-wise classification accuracy of approximately 96% against a rigorous manual ground-truth mask. The application of Sparse PCA was critical in isolating chemically diagnostic spectral features (480–600 nm) while effectively suppressing noise, thereby enabling robust spatial mapping even at crystal boundaries. Furthermore, the model’s generalization capability was confirmed through the evaluation of an independent, uncontaminated sample, which yielded zero false positives. These findings demonstrate that the developed workflow serves as a scalable, interpretable, and highly accurate screening tool suitable for supporting proactive contamination monitoring in the food industry.

## Data Availability

The authors stated and declare that all the datasets used and/or analyzed during the current study are available from the corresponding author on reasonable request to preserve the copyright. The authors stated and declare that all code exists and is available.

## Code availability

The authors stated and declare that all code exists and is available.
